# Order in Chaos: Lesser-Conserved and Repeat Structures in Dehydrins

**DOI:** 10.3390/biom15010137

**Published:** 2025-01-16

**Authors:** G. Richard Strimbeck

**Affiliations:** Department of Biology, Norwegian University of Science and Technology, 7491 Trondheim, Norway; richard.strimbeck@ntnu.no

**Keywords:** plant stress tolerance, intrinsically disordered protein, late embryogenesis abundant, tandem repeat, reticulon

## Abstract

Dehydrins (Dhns) are a group of intrinsically disordered land plant proteins that are closely associated with tolerance of dehydrative stress. Dhns are recognized and classified by the presence and sequence of five different conserved segments, varying in length from 8 to 15 residues, separated by highly variable disordered regions. In addition to one or more copies of the diagnostic, fifteen-residue K segment, most Dhns can be classified into one of three major groups based on the mutually exclusive presence of three other conserved segments (H, Y, or F), with all three groups typically incorporating multi-serine S segments. Many Dhns also include repeat structures. From an input library of 8675 non-redundant candidate sequences, a specialized R script identified and classified 2658 complete and 236 partial Dhn sequences in all major green plant (Viridiplantae) lineages, including a few green algal genera. An examination of the connecting segments bridging the conserved segments identified additional conserved patterns, suggesting that multi-Y, S-K, and K-S domains may act as functional units. Dhn Decoder identified 857 Dhns with repeat structures, ranging from 3 short, simple repeats to elaborate variations with up to 45 repeats or repeats of up to 85 residues comprising 1 or more of the conserved segments, suggesting that internal sequence duplication is an important mode of evolution in Dhns.

## 1. Introduction

Dehydrins (Dhns) are a family of intrinsically disordered plant proteins that are closely associated with the tolerance and survival of dehydration, freezing, and other dehydrative stresses. They are classified as a subset of late embryogenesis abundant (LEA) proteins that were first discovered and described in orthodox (dehydration tolerant) seeds [1]. Dhns were first named and had their general characteristics described over 25 years ago [2,3]. They have been found in all major land plant groups from bryophytes to angiosperm crown groups, and in most if not all plant tissues from roots and seeds to buds and leaves. They are highly hydrophilic, enriched in glycine and polar residues, heat stable, and largely unstructured in aqueous solution, as indicated by CD spectra. Some are constitutively expressed, but expression may increase in response to stress, while others seem to be produced mainly in response to specific stresses [4]. There is now an extensive body of scientific literature on Dhn occurrence, localization, response to stress, and, to a lesser extent, function [5,6,7,8,9].

The central identifying feature of Dhns is the 15-residue K segment, with the consensus sequence, usually given as EKKGIMDKIKEKLPG, that occurs in anywhere from 1 to as many as 15 copies and is generally well-conserved in amino acid identity or properties across the entire spectrum of land plants. In a nonpolar environment, the K segment folds into an amphipathic a-helix. K segments have been shown to bind to membranes, suggesting that Dhns help prevent dehydration-induced denaturation of these critical structures [10,11].

While some Dhns contain only one or more K segments, the following four other conserved segment types may occur in various arrangements in different subgroups of Dhns:

• Y segments, originally described by the 7-residue consensus sequence (V/T)DEYGNP [3], typically occur in 1 to 4 copies N-terminal to other conserved segments, but more uncommonly in as many as 36 copies and rarely between other conserved segments. Melgar and Zelada [12] recently noted that the Y segment consensus can be extended to include an eighth, nonpolar residue, usually V or I.
• F segments, first described in 2017 [13] and confirmed in a MEME analysis of 426 Dhns in 53 angiosperm and 3 gymnosperm genomes [14], vary around the conserved core sequence DRGLFDFLGKK and typically occur in a single copy N terminal to other conserved segments. An uncommon variant, GCGMFDFLKK, here called the GC-variant, is known to occur only in rosid and a few asterid species [13].
• H segments, first described in 2021 [12], with the 15-residue consensus M(A/S)GIIHKIEE(T/K/A)LH(I/M)G, occur at the N terminus of Dhns with a terminal S segment.
• S segments consist of strings of 3 to 13 serine residues, typically interrupted by 1 or a few single D or E residues, with additional conserved prefix or suffix strings that vary somewhat in different types of Dhns [13]. They typically occur in one of two positions, either between Y or F segments and K segments or at the C terminus of the Dhns with N-terminal H segments [12].

Based on the number and position of the five conserved segment types, Dhns are classified into different groups, with new variants added to the list with the recent discoveries of the F and H segments. The common types are Kn, SKn, HKnS, YnKn, YnSKn, and FSKn, with any other arrangements being relatively uncommon. A phylogenetic analysis of 305 Dhn amino acid sequences from 56 land plant genomes shows that Dhns fall into 3 orthologous groups [12], designated F-, H-, or Y-Dhns, here called Dhn orders and named F(S)Kn, (H)KnS, and Yn(S)Kn, respectively, with parentheses referring to the absence of diagnostic segments in some individual Dhns. Some Kn and SKn Dhns can be classified into one of these orders by phylogenetic analysis [12].

The conserved segments in Dhns are separated by highly variable strings of as few as two to typically tens but occasionally up to a few hundred residues, which together with N- and C-terminal strings are collectively referred to as phi segments. In keeping with the hydrophobic nature of Dhns, these are usually rich in glycine and polar residues.

Due to their lack of a secondary structure and conservation outside of the conserved segments, Dhns are classified as intrinsically disordered proteins [7]. However, on further examination and analysis, some additional, less-conserved features appear that may provide additional clues for investigation of Dhn diversification and function, such as the S-K segment described by Malik et al. [15]. Among these details are repeat structures, strings of amino acids that are identical or highly similar within the same protein, but which are not conserved over phylogenetic distance. These become evident on close examination of some Dhn amino acid sequences, and apparently arise as a result of internal duplication within a gene [16,17]. Repeats may occur only in phi segments or may include one or more of the conserved segment types. Repeats are common features in many proteins, ranging from short and disordered to longer stretches that contribute to the secondary structure and functionality, for example in transmembrane domains [17], but they are especially common in intrinsically disordered proteins [16]. Their occurrence in Dhns suggests that they have played a role in Dhn diversification and possibly in the evolution of Dhn function.

To explore the diversity of Dhn structure and the occurrence of repeat sequences in Dhns, I developed a specialized R script, dubbed Dhn Decoder, to scan candidate sequences containing K-like segments for all five types of conserved segments, classify the results according to the number and order of conserved segments, and search for and report on repeated sequences. The resulting library, while not necessarily comprehensive, includes numerous sequences not previously identified as Dhns and offers insight into conservation and variation in Dhn structure, the taxonomic occurrence of different Dhn types, and the role of internal sequence duplication in Dhn diversification. This new information may help guide studies of Dhn function and evolution.

## 2. Materials and Methods

To obtain a library of candidate Dhn sequences, I used BLASTp to search the NCBI clustered database for variations on the K segment consensus sequence (EKKGIMDKIKEKLPG) and downloaded the top 5000 hits as of October 2024, using default BLAST settings and limiting the search to Viridiplantae. After finding that HKn(S) Dhns have a distinct variant of the K segment (consensus HKEGFVDKIKDKIHG, discussed below), I added the top 5000 hits from a search on this sequence, then filtered out the second copy of duplicate sequences that were found in both searches.

I developed an R script (version 4.3.2, R Foundation for Statistical Computing, Vienna, Austria), Dhn Decoder (Supplementary File S1), that scans candidate sequences for the five conserved segments and for repeat strings of a specified length. Libraries of previously identified K, Y, F, H, and S segments were used to develop frequency tables of amino acid residues at each position in the segment, using 15, 8, 10, 15, and a variable number of positions, respectively, with separate tables for the canonical and HKn(S) variants of the K segment. To facilitate identification of GC-variant F segments, the frequencies for G and C in the first two positions, respectively, were set to the same values as for D and R in the same positions. The frequency tables were used to score candidate segments for overall conservation. Individual amino acid sequences were scanned for H, Y, F, S, and K segments in that order, one starting position at a time. Because of their variable length, S segments were tentatively identified by scoring the first five residues and extended until no two consecutive residues were an S or upon reaching the C terminus for C-terminal S segments. A final score, adjusted for segment length, was calculated using a table with frequencies for the first 5, a variable number of intermediate, and the last 2 residues in the segment. Threshold scores for each segment type were determined by examining the results of initial runs. These are necessarily somewhat arbitrary but were selected to include variants of the canonical segments and exclude more spurious variants, with the risk of missing some rare but possibly functional variants. A running count of identified segments in each sequence was used to produce a structural formula in the HFYnSKnS format.

Sequences with any segments over the threshold value were then scanned for 6 or 10 residue repeats, with an 80% identity requirement for a string to be recognized as a repeat, and at least 3 copies required to be recognized as a putative repeat sequence.

The results were summarized in the following four files:

1. The DhnTypes file lists all sequences containing any K segments scoring over threshold values together with a structural formula.
2. The Segments file lists all identified segments by NCBI accession, score, and sequence, including the phi segment preceding each identified segment and any C terminal segment following the last conserved segment, usually K or S. For brevity, phi segments are referred to as N-terminal, C-terminal, or by the enclosing conserved segments, for example Y-Y or S-K. This was used to compile lists of these different types of phi segments to assess variation and conservation in these transitional segments.
3. The Repeat Summary file lists the number and sequence of repeat strings by accession.
4. The Repeats file gives a preliminary parsing of sequences found to contain three or more repeat strings.

For inspection and further analysis, all four output files were assembled as separate worksheets in a Microsoft Excel workbook (Supplementary File S2), with an additional worksheet containing all candidate sequences. Based on the structural formula and further inspection, sequences were assigned to one of thirteen groups: K1, Kn, SKn, KnS, HKn, HKnS, FKn, FSKn, YnKn, YnSKn, irregular (other arrangements), pseudo (K1 and SKn Dhns with low-scoring K segments and little resemblance to other known Dhns), and reticulon (discussed below). Irregular Dhns were manually checked for low-scoring segments and sometimes reclassified as one of the main types by ignoring low-scoring segments. The last two groups are provisionally considered non-Dhns but include some proteins with Dhn-like characteristics that may be worth exploring in the future. Columns for species name and description (from the NCBI listing), genus, family, and major taxonomic group (Chlorophyta, Bryophyte, Pteridophyte, Gymnosperm, Basal angiosperm, Magnoliid, Monocot, Basal eudicot, Carophyllid, Rosid, Asterid) were added to the DhnTypes sheet, which was then used as a lookup table to transfer information to other worksheets. These columns were used to sort and search the rows on each worksheet.

Based on the phylogenetic analysis of Melgar and Zelada (2021), sequences in the eleven groups confirmed as Dhns, based on the presence of one or more conserved K segments, that also included F, H, Y, or C-terminal S segments, and including irregular Dhns, were assigned to the F(S)Kn, Yn(S)Kn, or (H)KnS orders, respectively, with the relatively uncommon KnS Dhns included in the latter group. Kn (including K1), SKn, and pseudo Dhns remained as independent groups.

Color annotation of conserved segments and rearrangements to document repeat sequences were performed manually on a copy of the Repeats worksheet entitled “Parsed repeats”.

Amino acid composition and molecular weights for all confirmed and complete Dhn sequences were extracted using the protr package in R [18]. Linear discriminant analysis (LDA; MASS package in R [19]) was used to differentiate first five groups, then the three described orders based on amino acid frequencies.

The conservation of amino acid identity or properties in Y and K segments and Y-Y, S-K, and K-S phi segments in different Dhn groups was evaluated by plotting amino acid frequencies by position after the last residue in the N-terminal enclosing segment, using colors based on the Kyte–Doolittle hydrophobicity to emphasize the conservation of properties.

## 3. Results

### 3.1. Verification

Dhn Decoder successfully identified all previously known Dhns in five land plant genera in the Phytozome database, namely *Selaginella*, *Amborella*, *Aquilegia*, *Oryza*, and *Arabidopsis*, as well as some additional sequences not previously identified as Dhns, and some partial sequences and significant variants of known Dhns which may be of interest in studies of Dhn evolution or function (Supplementary File S2). This demonstrates that the script is a useful tool for finding Dhns in input amino acid sequences.

### 3.2. Dhn Groups and Orders

The BLASTp searches on the 2 variants of the K segment yielded 8675 unique candidate sequences (candidate sequences csv file, Supplementary File S1). After the initial assessment of the results with lower threshold values, the threshold scores for the recognition of K, H, F, Y, and S segments were set at 6.0, 6.8, 5.0, 3.5, and 5.2, respectively. Using these values, the Dhn Decoder R script identified K-like segments in 4044 sequences, including 293 designated as partial according to the NCBI description or by lack of an N-terminal M residue. A total of 1142 sequences were reclassified as pseudo Dhns or reticulons (discussed below), leaving 2658 complete and 236 partial sequences with well-conserved K segments broadly distributed across green plant groups (Table 1). The resulting list still includes some likely paralogues or allelic variants in some taxa, often differing by indels of sufficient length to result in <90% identity, and it may exclude some known Dhns with >90% identity with Dhns in the final list.

The K1, Kn, KnS, and irregular groups included 982 sequences with 1 or 2 single, relatively weakly conserved K segments, with consensus residues in some positions but others not conforming to type. Because these weak segments leave some doubt as to whether these are functional Dhns, all Dhns in these four groups with maximum K segment scores <7.0 were given a provisional status as pseudo Dhns. After this screening, the remaining K1 Dhns were folded into the Kn group in further analysis.

The search algorithm also identified a group of 160 KS sequences with a fused KS-like segment (e.g., KEESLMEKIKEKLHGDSSSSDSD, NCBI accession XP_039115618.1, *Dioscorea cayensis*) near the N-terminus rather than with a C-terminal S segment as in typical (H)KnS Dhns, and with the K segment portion usually scoring < 7.0. Some of these were tagged as reticulons in the NCBI database. Known Arabidopsis reticulons contain a similar KS-like segment near the N-terminus [20]. Most of these were already identified in NCBI as reticulons or reticulon-like; additional putative reticulons were identified manually by screening all KS DHNs identified by the script for lack of a terminal S segment and then checking for the presence of the fused KS-like segment near the N terminus. These sequences are well-conserved across a broad range of land plants (Figure S1). Sequences reclassified as reticulons and pseudo DHNs were excluded from further analysis but included in the database because they may be of future interest. This raises the possibility that anti-K segment antibodies may bind to reticulons and the low-scoring K segments in pseudo Dhns, resulting in misidentification of these proteins as Dhns using immunological methods. Although eukaryote reticulons are recognized by a conserved reticulon homology domain at the C-terminus (Figure S1), it is noteworthy and perhaps intriguing that plant reticulons have this Dhn-like characteristic.

Dhn Decoder identified 69 GC-variant F segments among the 620 F(S)Kn Dhns, with 17 in asterid species and the remainder in rosids.

Among the 2658 complete and confirmed Dhn sequences, Yn(S)Kn Dhns were most common at about 36% of complete sequences, followed by F(S)Kn Dhns at about 22%. (H)KnS and Kn Dhns comprised about 9 and 1% of the complete sequences, respectively, with SKn Dhns comprising about 17% and Kn about 14% (Table 1), leaving 2% classified as irregular.

**Figure 1:**
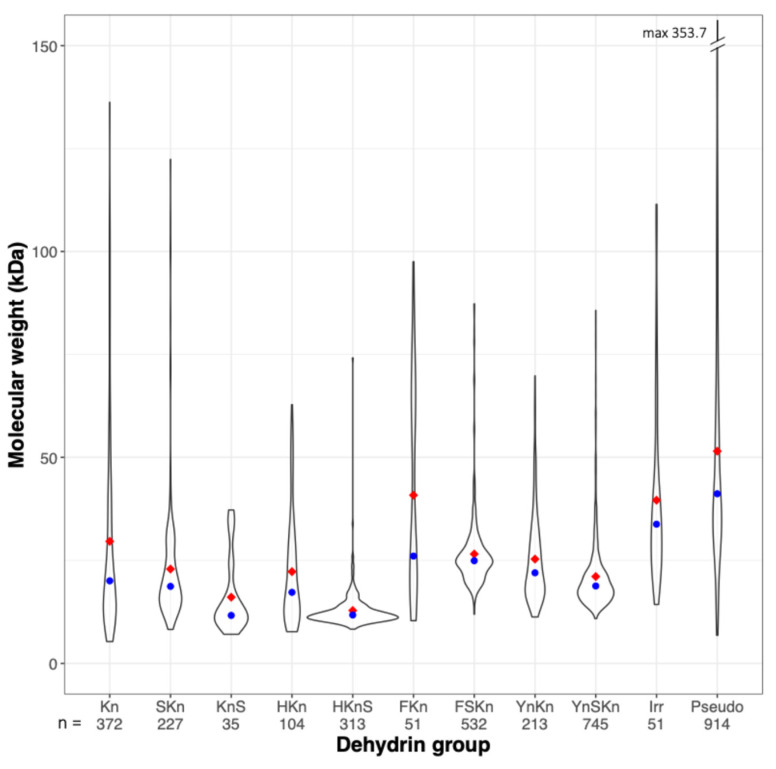
Violin plot of the molecular weight distributions of Dhns by group. The red diamond indicates group mean and blue dot indicates median.

Excluding pseudo Dhns, complete Dhn sequences varied in length from 55 to about 1140 residues and molecular weight varied from 4.7 to 183.8 kDa. Molecular weight distributions for the different Dhn groups are skewed towards lower weights (Figure 1), with median values below 25 kDa in all groups except the FKn group, which includes 14 Dhns with 5–11 K segments in *Salix* and *Populus* and 5 unusual F2-3K3 Dhns in *Quercus* and *Castanea*, all with molecular weights >50 kDa, and the irregular group, which is comprised mainly of larger, compound Dhns, as discussed below. As noted by Melgar and Zelada [12], HKnS DHns are the lowest-weight Dhn group, with a narrow molecular weight distribution centered on a median of about 12 kDa. The pseudo group includes 26 sequences with MW > 150 kDa and numerous others with relatively high MW, another characteristic which differentiates them from known Dhns.

### 3.3. Taxonomic Distribution

Dhn Decoder identified nine sequences with K segments scoring above the threshold in green algae (Table 1), in six genera, including *Chlorella*, *Scandesmus*, *Klebsormidium*, and the lichen symbiont genus *Symbiochloris*. Most were described as a “hypothetical protein” in NCBI. The single K segment in 20 additional algal sequences over a broad range of genera and families scored below 7.0, resulting in their reclassification as pseudo Dhns.

The 27 bryophyte Dhns identified by the algorithm include 18 Kn and 9 YnKn or irregular Dhns with 1 or more low-scoring Y segments. Yn(S)Kn Dhns were otherwise found only in angiosperms. In addition, 13 bryophyte sequences were reclassified as pseudo Dhns based on low K segment scores.

Lycophytes and ferns are underrepresented but include three and two SKn and (H)KnS Dhns, respectively. No Y segments were found in these groups or in gymnosperms, and F(S)Kn Dhns were found only in seed plants. Green algae, ferns, lycophytes, and gymnosperms accounted for only about 5% of the total number of complete sequences, so further investigation might turn up other Dhn types in these groups.

### 3.4. Dhn Groups Differ in Amino Acid Composition

**Figure 2:**
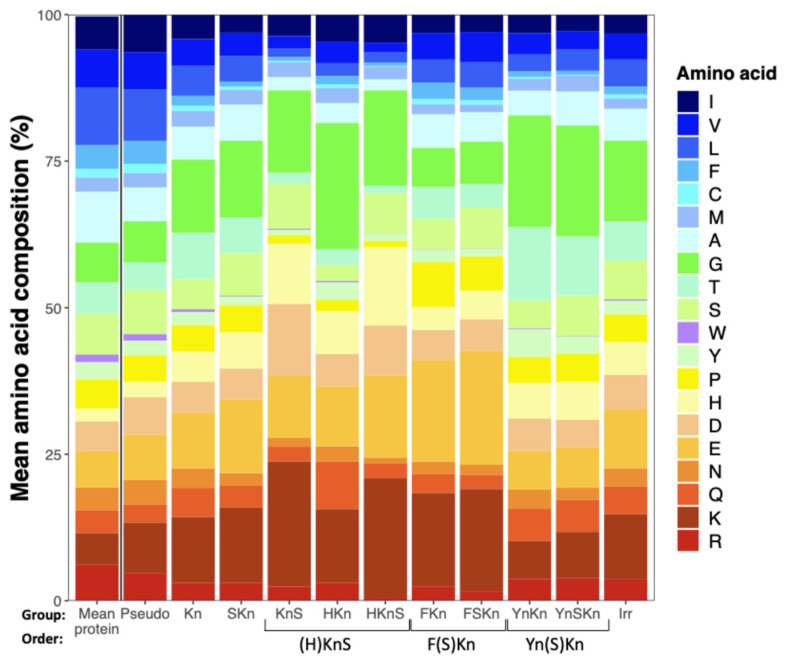
Amino acid composition of Dhn groups compared to average proteins. Amino acids are colored and ordered by Kyte–Doolittle hydrophobicity.

As compared to the mean amino acid composition of a large sample of protein sequences [21], Dhns are lower in hydrophobic residues and generally enriched in charged amino acids, histidine, and glycine (Figure 2). Comparing Dhn groups, those with F segments are higher in charged amino acids and lower in Gly, while those with Y segments follow an opposite pattern and have relatively high levels of Thr and Tyr, both of which appear in Y segments. (H)KnS Dhns have similar proportions of Ser to other groups, but are enriched in His, with the HKnS group having the lowest proportion of hydrophobic amino acids of any group. Pseudo Dhns have a higher proportion of hydrophobic amino acids than all other groups and a relatively low proportion of Gly, more closely resembling the mean amino acid composition of average proteins.

**Figure 3:**
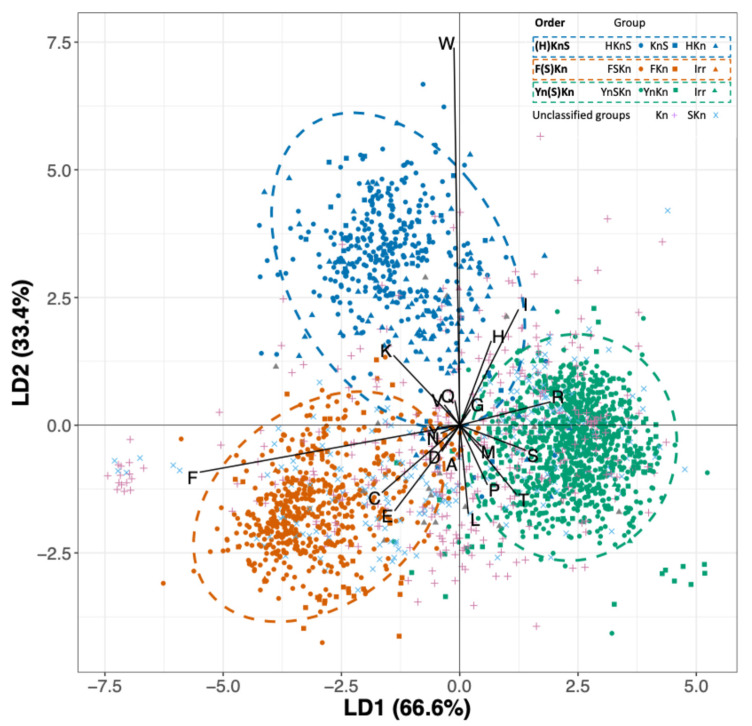
Linear discriminant analysis of amino acid composition of Dhns assigned to three well-defined orders, with unassigned Dhns added using the LDA function.

A five-group linear discriminant analysis on amino acid composition, including the three Dhn orders plus Kn and Skn as separate groups (excluding pseudo Dhns and with Irr Dhns assigned to orders according to whether they have F or Y segments; none had H segments), gave an approximately 77% predictive accuracy, with >80% accuracy for the three phylogenetically defined orders but weak discrimination of the unclassified Dhns. Excluding Kn and SKns Dhns and reducing the model to just the three orders gave about 97% overall predictive accuracy, with accuracy ranging from 94% for (H)KnS to >99% for Yn(S)Kn Dhns (Figure 3). Thus, Dhns in the three phylogenetically defined orders can be differentiated based on their amino acid composition. Factor loadings indicate that (H)KnS-Dhns have relatively high proportions of Trp and somewhat higher levels of Lys, His, and Ile, F(S)Kn Dhns are enriched in Phe and somewhat in Cys and Glu, while Yn(S)Kn Dhns have slightly higher amounts of various amino acids including Thr as compared to the other two groups.

The LDA model prediction distributed Kn and SKn Dhns among all three orders, suggesting that many of these may have evolved by deletion of H, F, or Y segments. The outlying cluster of Kn Dhns with LD1 values < −6, mainly in Asteraceae, are enriched in Lys and Glu (e.g., KVI07719.1 *Cynara cardunculus* var. *scolymus*, Supplementary File S3), likely due to the internal duplication of short segments enriched in these two amino acids.

### 3.5. H, F and S Segments Almost Always Occur Singly

H segments were initially described as occurring singly at the N terminus of the protein [12]. However, Dehydrin Decoder identified 15 proteins with phi segments of mostly <100 residues (max 527) preceding the H segment, and 3 sequences with more than 1 H segment (i.e., KAF9670274.1, HK2HK2), leaving about 96% of HKnS Dhns adhering to the original description. Sequences with a single K segment (HK and HKS) were most common, comprising about 64% of (H)KnS Dhns.

Of the 595 complete F(S)Kn Dhns identified by Dehydrin Decoder, 20 (3.5%) have more than 1 F segment, with a maximum of 8 in an F8SK3 Dhn (XP_050215137.1, *Mercurialis annua*), one with 5, and the remainder with 2 or 3. Multiple S segments occur only in the 19 irregular Dhns (about 1% of Dhns with any S segments), and then never in tandem. In contrast, K and Y segments may occur in as many as 15 and 36 copies, respectively, with the latter often being closely spaced (e.g., AAL83427.1, *Cornus sericea*, Supplementary File S3) [22]. This suggests that there is some functional constraint on the number and arrangement of H, F, and S segments in Dhn evolution.

### 3.6. Y Segments Comprise Eight Conserved Residues and Are Closely Spaced

The Y segment was initially described as a seven-residue conserved sequence with the consensus formula (V/T)DEYGNP [3]. Until recently, this description was accepted throughout the Dhn literature (e.g., [15]), but Melgar and Zelada [12] noted that the conserved segment can be extended by addition of a hydrophobic residue, usually Val or Ile, at the C terminal end, giving the updated consensus sequence TDEYGNP(V/I). The library of about 2000 Y segments identified by Dhn Decoder confirms this observation (Supplementary Figure S2). An 8-residue scoring table based on this version of the Y segment was used to identify and score Y segments in the final run of Dhn Decoder.

**Figure 4:**
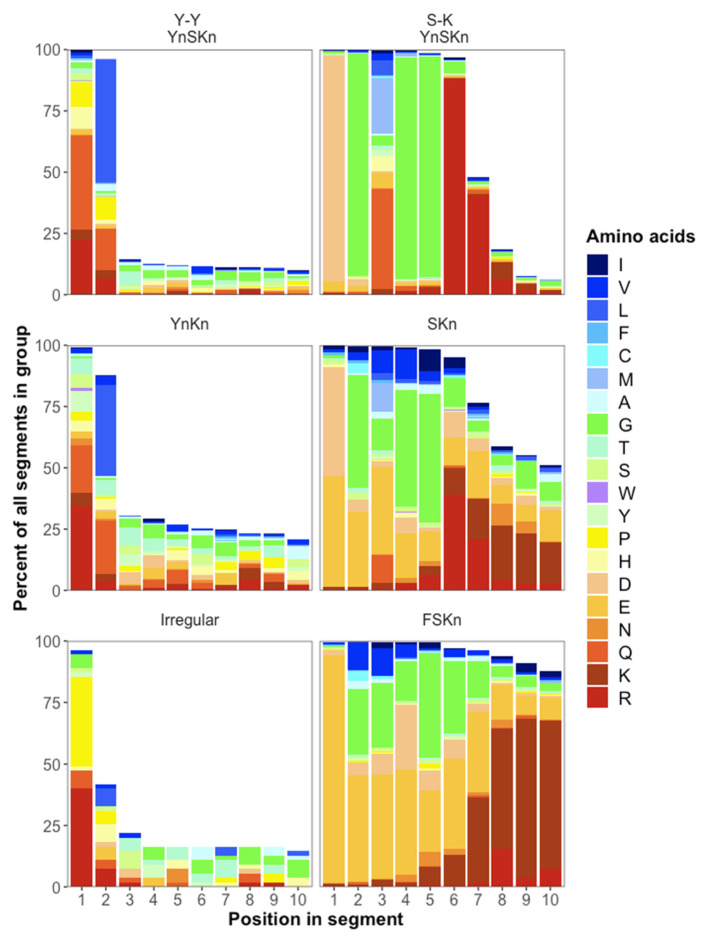
Amino acid frequencies in up to 10 residues in phi segments between paired Y segments (Y-Y) or S and K segments (S-K). Blanks occur when the phi segment is shorter than 10 residues.

It is evident upon casual inspection that multiple Y segments tend to be closely spaced. About 85% of 898 Y-Y linkages in YnSKn Dhns consist of just one or two residues, with the first usually being polar and second typically another polar residue or leucine (Figure 4). In YnKn Dhns, about 10% of paired Y segments are separated by a single residue, with another ca. 60% separated by 2 residues, and about 25% comprising 10 or more residues. In the small number of irregular Dhns with multiple Y segments, about 60% are separated by a single residue, usually Arg or Pro, with another ca. 20% including a second variable residue, while about 15% are separated by 10 or more residues. Many of the longer Y-Y phi segments occur in Dhns from with 4 to as many as 36 Y segments, apparently generated by internal repeats that include more extended Y-Y segments that may account for the greater separation. This consistently close spacing of multiple Y segments suggest that these domains may function as a unit.

### 3.7. K Segments in (H)KnS Dhns Differ from Those in Other Dhns

**Figure 5:**
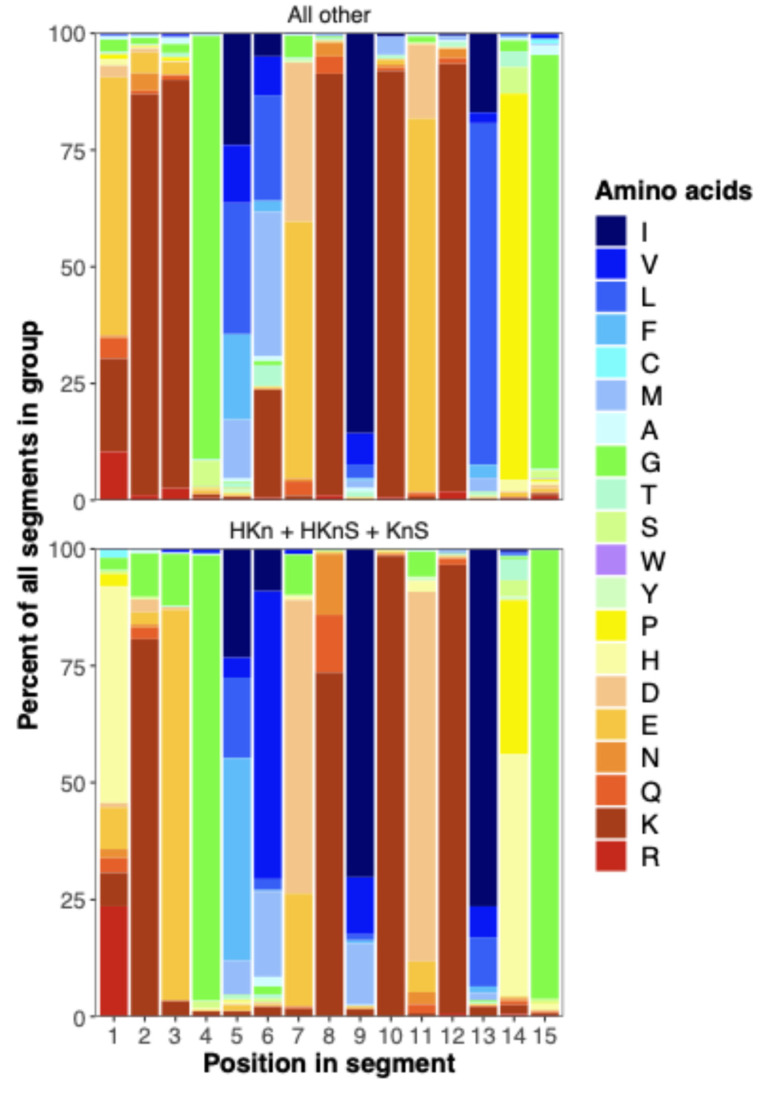
Amino acid frequencies in K segments in (H)KnS Dhns compared to other Dhn groups.

Comparison of K segment amino acid frequencies in (H)KnS Dhns versus other groups reveals minor but consistent differences, with the variant consensus sequence HKEGFVDKIKDKIHG distinguishing the former group (Figure 5). The substitution of His for a charged residue in position 1 in about 40% and for Pro in position 14 in about 50% of (H)KnS Dhns may affect the membrane binding or other properties of the protein via reversible protonation of His residues [11]. Another, perhaps more minor, difference is the substitution of an acidic for a basic residue at position three.

### 3.8. K Segments Are More Conserved Toward the C Terminus

Anecdotal observation of K segment sequences at different positions in the protein suggested that K segments are more conserved toward the C terminus of the protein (Figure S3). K segments nearer to the C terminus score higher than N-terminal or intermediate segments in most F(S)Kn and Yn(S)Kn Dhns (about 62 and 82%, respectively) with two or more K segments. Most Kn and (H)KnS Dhns have only a single K segment, and intermediate K segments frequently score higher in Dhns with two or more K segments in the latter group.

C-terminal K segments tend to be located close to the C terminus; among all Dhns lacking a terminal S segment, about 82% of C-terminal K segments are located within 16 residues of the C terminus, with 64% within 8 residues (Figure S4). K segments are located within 2–8 residues of the C terminus in about 87% of Yn(S)Kn Dhns, but C-terminal phi segments tend to be longer in F(S)Kn Dhns, with about 88% in the 9–32 residue length classes. C-terminal segments are more evenly distributed in Kn and SKn Dhns, with 79% and 91% in the 2–32 length classes, respectively.

### 3.9. K Segments Are Often Flanked by His Residues

Eriksson et al. [11] found that pH-dependent protonation of His residues affects the membrane binding behavior of Arabidopsis Lti30, a K6 Dhn with paired His residues flanking five K segments, with the remaining N-terminal segment also followed by a pair of His residues. While this specific arrangement is not widely conserved, Dhns are enriched in His compared to average proteins (Figure 2). I tallied H residues in 6-residue prefixes and suffixes (or as few as one residue in K segments ending within 6 residues of the C terminus) of the 6420 K segments in the 2893 complete and partial Dhns identified by Dhn Decoder (Table S1). Overall, about 77% and 42% of all K segments had at least one or two H residues, respectively, in close proximity, with these proportions at 87% and 58% for K segments located toward the C terminus of the protein, and generally higher proportions of His on the C terminal (suffix) side than the N terminal (prefix) side. Flanking H residues are most abundant in F(S)Kn and Y(S)Kn Dhns, with at least one His flanking >90% of C terminal K segments in both groups, and are somewhat less abundant in other groups, including (H)KnS Dhns, which typically include H residues within the K segment (Table S1). While not universal, these high proportions together with the previous work suggest that pH-dependent modulation of membrane binding behavior via reversible protonation of His residues in close proximity to K segments may be a general property of Dhns.

### 3.10. S-Phi-K and K-Phi-S Regions Are Variably Conserved in Length and Composition

Malik et al. [15] described a ca. eight-residue S-K segment beginning after the last acidic residue in the S segment and consisting mainly of Gly and positively charged Lys and Arg residues. However, S-K linkages vary in length and composition between Dhn types (Figure 5). About 80% of the 371 S-K phi linkages recorded for YnSKn Dhns consist of six or seven residues conforming to the consensus sequence DGXGGR(R), where X is about 50% Gln or Glu, but may be a hydrophobic residue in about 30% of the linkages (Figure 5). Longer variations comprise additional Arg, Lys, Gly, Asp, or Glu residues. S-K phi segments in SKn and FSKn Dhns are less strongly conserved but generally consist of a mix of Glu, Arg, Gly, and Lys residues, with small proportions of hydrophobic residues (Figure 5).

With few exceptions, S segments occur at the C-terminus of (H)KnS Dhns, linked to a K segment by a K-S linkage that varies in length between 18 and 46 residues in about 90% of the 374 K-S linkages in (H)KnS Dhns identified by Dhn Decoder. While difficult to describe in a simple formula and so here presented as supplementary data (Supplementary Table S2), these can be divided into three domains, namely a GDE(H) domain with a mix of Gly, Asp, Glu, and sometimes His residues; a poly-K string occasionally interrupted by Glu or Asp; and a second GDEH string with one to five His residues. A more limited version of this linkage has been described as a conserved, K-rich B segment [12].

### 3.11. Internal Repeats Are an Important Mode of Dhn Diversification

**Figure 6:**
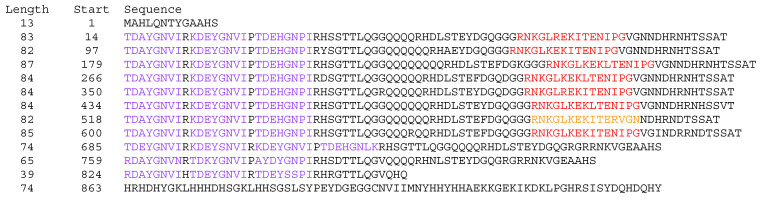
Complete sequence of a *Juglans macrocarpa* × *Juglans regia* YnKn Dhn, (NCBI accession XP_041020672.1, “mesocentin-like isoform X2”), with the complex formula (Y3K)_8_Y4(Y3)_2_, showing the alignment of internal repeats. Color coding: purple, conserved Y segment; red, conserved K segment; orange, low-scoring K segment inferred by position.

The Dhn Decoder script identified at least 3 6- or 10- residue repeats with a minimum 80% identity in 857 of the 2894 complete or partial proteins recognized as Dhns by the presence of 1 or more conserved K segments, with repeat structures identified in an additional 145 Pseudo Dhns. Repeat numbers identified by the algorithm ranged from the specified minimum of 3 to 45 in a Glu- and Lys-rich K1 Dhn in *Cynara cardunculus* (NCBI accession KVI07719.1; the repeat-aligned sequences of this and other Dhns noted below are given in Supplementary File S4). Repeats are trivial in some sequences, with a low number of short repeats, but in most cases can be extended to a maximum of about 85 residues (i.e., XP_041020672.1 and its orthologues or alleles in *Juglans* spp., Figure 6) duplicated with sufficient fidelity to infer internal duplication within the gene, often with substitutions or indels.

Repeats may occur in tandem, for example in a *Prunus avium* Y2K9 Dhn (XP_021833744.1), or separated by strings that suggest insertion of the repeat sequence at different locations in a preexisting sequence. In the F2K2 *Carpinus fangiana* Dhn KAE8057341.1, the repeat structure can be characterized as “variations on a theme”, with 35 repeats in 4 different variants of 10, 11, 16, and 21 residues, all based on the consensus sequence GYHKEEPK. These appear to be shuffled in no recognizable order but were presumably produced by a complex series of internal duplications and insertions. A similar architecture consisting of 11- and 18-residue Glu- and Lys-rich repeats occurs in a group of K1 Dhns in at least 6 genera in 5 different tribes in Asteraceae exemplified by the 45-repeat *Cynara cardunculus* noted above (KVI07719.1), with a probable orthologue in *Daucus* (XP_017252260.1).

Importantly, longer repeats may contain conserved segments, usually Y or K, providing a mechanism for the proliferation of functional segments in Dhns. In the irregular *Juglans macrocarpa* x *J. regia* Dhn noted above (XP_041020672.1, Figure 6), there are eight Y3K repeats followed by a Y4 and two Y3 variants. This architecture gives rise to the complex formula (Y3K)_8_Y4(Y3)_2_, for a total of 34 Y segments and 8 K segments. In many cases, the alignment of repeats containing conserved segments allows the recognition of additional, low-scoring segments of the same type at identical or similar positions in the repeat. While these may be interpreted as degraded and perhaps non-functional versions of the segment type, I will refer to them as altered or low-scoring. For the purposes of reporting, I include these inferred segments when describing segment counts and architecture, but it is important to note that they are given distinct colors in Supplementary File S3.

In some cases, proteins in related taxa have different numbers of similar repeats. Orthologs of the irregular Dhn in *Juglans macrocarpa x regia* described above have a similar architecture but with three to eight instead of eleven repeats and, therefore, fewer total Y and K segments. A group of FKn Dhns in *Salix* and *Populus* species have from 6 to 14 K segments contained in 2 main repeat variants of length 29 to 46 residues (e.g., the FK14 ADL59574.1, *P. alba x glandulosa*). In the unusual Y36SK2 Dhn in *Cornus sericea* (AAL83426.1), the 36 Y segments (15 inferred by position despite low conservation) are contained in 12 copies of a highly regular Y3 repeat. Additionally, 2 variants in the same species (AAL83427.1 and AAL83428.1), contain 26 and 28 Y segments, respectively, with the insertion of a fourth Y segment in some repeats resulting in less regular architectures.

## 4. Discussion

### 4.1. Strengths and Limitations of the Dhn Decoder Script

The main value of Dhn Decoder is that it casts a wide net in searching for putative Dhns, relying primarily on finding K-like segments based on the strong conservation of amino acid identity or properties in the canonical and H-type variants of the segment across a broad range of land plant species. Further identification of H, F, and Y segments, where present, can be used to place Dhns in the main Dhn orders. The threshold values for each segment type can be changed for more or less stringent identification of all segment types. The structural formulas help further classify and characterize Dhn types. Identification of repeat structures may be of interest in studies of evolution in Dhns and other intrinsically disordered proteins.

As demonstrated by my reclassification of many sequences as pseudo Dhns or reticulons, curation of the Dhn Decoder output is necessary to filter and confirm the results, especially for sequences lacking H, F, or Y segments. The database may still include non-Dhns with one or a few weak, K-like segments, but the presence of H, F, and Y segments in most sequences helps confirm their identity as Dhns in the respective orders.

The script does not take into consideration phylogenetic relationships among Dhns; that is left for future work. Even after clustering based on 90% identity in the NCBI non-redundant protein database, the results include some allelic or ecotypic variants that may be of some interest in future research. With some curation of the results, researchers interested in Dhns in specific plant taxa should be able to use the script to search for and classify all or most Dhn-like sequences in non-clustered sequence data for a more complete picture of Dhn variation within a taxon, including among ecotypes or agricultural strains that vary in stress tolerance.

### 4.2. Dehydrins in Early Land Plant Evolution

Herein is, as far as I know, the first report of confirmed Dhn sequences in green algae. None of the green algal sequences identified by Dehydrin Decoder included H, F, or Y segments, all of which appear to have evolved later in land plant evolution. One SK1 Dhn and one SK2 Dhn were found in *Klebsormidium nitens*, a filamentous alga that occurs in terrestrial environments, including desert soil crusts subject to severe desiccation, suggesting that the S segment may have evolved during adaptation to terrestrial or periodically dry environments. The pseudo K1 and K2 Dhns in *Symbiochloris* and the additional K12 Dhn in *Myrmecia* are of interest because these genera represent the main family of lichen photosymbionts, the Trebouxiaceae, wherein tolerance of rapid cycles of desiccation and rehydration are part of the lifestyle. Gasulla et al. [23] provided immunological evidence of Dhns in the lichen symbiont *Trebouxia erici*, but this seems to be the first report of a confirmed Dhn sequences for lichen photobionts.

The K segments in the Kn algal Dhns (i.e., the C-terminal K segment in the *Klebsormidium* SK2 Dhn GAQ85800.1, EKKGFFKKIEEKLTG, score 8.35), generally conserve the identity or properties of the residues in the canonical K segment land plant Dhns. BLASTp searches on the full sequences of the algal Kn Dhns did not return any strong alignments with bryophyte or other land plant Dhns, perhaps not surprising given the evolutionary distance between these groups and the unstructured and highly variable character of Dhns. The majority of the green algal sequences were classified as pseudo Dhns with single, low scoring variants of the K segment. Allowing for modification of the K segment in land plant evolution, some or all of these may be functional Dhns with ancestral characteristics.

Dhn-like proteins have been detected immunologically in brown rockweeds [24] and cyanobacteria [25]. My search for candidate sequences in NCBI was restricted to Viridiplantae, thereby excluding cyanobacteria, red algae, and heterokonts including brown algae. Provisional searches in NCBI for the canonical K segment sequence and the (H)KnS variant in cyanobacteria, heterokonts, and rhodophyta yielded only a single, tantalizing K segment variant, KHEGLMDKIKDKLPG, with a score of 8.3 in Dhn Decoder, in an unclassified protein (KAG5181971.1) in the genome of the freshwater, yellow-green (Xanthophyte) alga *Tribonema minus.* The sequence also includes three poly-S segments, all followed by a pair of Asp residues similar to those in Dhn S segments. *T. minus* can survive freezing and desiccation, including in polar environments, and is under evaluation for biodiesel fuel production and other potential applications [26,27]. Recognizable fragments of the K segment sequence, usually variations on KIKEK or GIMD, turned up in other algal species, but I found no other high-scoring full-length variants.

Y-like segments, ending with a charged rather than hydrophobic residue as in angiosperms, were noted in nine bryophyte Dhns, including a nine-repeat Y9K sequence (XP_024395993.1) in *Physcomitrium*, but otherwise have been found only in angiosperms. This raises the possibility that Y-like segments first evolved in bryophytes but were subsequently lost in branches leading to gymnosperms and perhaps lycophytes and pteridophytes. As with green algae, the K1 sequences classified as pseudo Dhns in the liverwort *Marchantia* may represent early or divergent versions of the K segment consensus sequence.

The occurrence of (H)SKn Dhns in lycophytes and ferns suggest that the H segment first appeared in early vascular plant evolution, but it is conspicuously missing in gymnosperms. F segments were noted in lycophytes, but not among the six Dhns found in ferns, and are common in gymnosperms and angiosperms. It seems likely that the F segment also evolved early in vascular plant evolution and that further investigation of fern genomes may turn up some F(S)Kn Dhns.

### 4.3. Dehydrin Orders

In something of a breakthrough in the Dhn world, a recent, ambitious phylogenetic analysis divided 307 Dhn sequences into 3 well-defined groups, corresponding to the (H)KnS, F(S)Kn, and Yn(S)Kn orders described here [12], including the discovery and description of the H segment. The authors also documented consistent differences in the pI, Gravy index, and fold index among the three Dhn orders. Their findings are supported by my LDA analysis of amino acid composition (Figure 3), which clearly delineates the three main groups, leaving about 600 Kn and SKn Dhns unclassified. A total of 15 of the 307 sequences in Melgar et al.’s analysis lacked H, F, or Y segments, but 1, 2, and 12 of these were placed in the above-named orders, respectively, many with > 90% bootstrap support, indicating a possible origin by deletion of an accessory segment. However, the known Dhns in algal groups lack any of these accessory segments and bryophytes lack H and F segments, suggesting that some Kn and SKn Dhns may represent basal Dhn types that evolved before the origin of additional segments.

Despite wide variability in sequence and length within and between taxa, each order can be characterized by a typical or consensus structure composed of a predictable order of conserved segments separated by somewhat more variable transitional regions with some conservation in amino acid composition and structure, and phi segments that are highly variable in length and composition.

Reading from the N to C terminus, a typical (H)KnS Dhn has the following segments:

• A single H segment at the N terminus.
• A random coil phi segment.
• Usually one K segment with the variant consensus HKEGFVDKIKDKIHG, separated by highly variable phi segments.
• A variable length transitional segment comprised of Gly and charged residues interrupted by a poly-K region and with His residues at the C-terminal or both ends.
• A single C-terminal S segment.

(H)KnS Dhns almost always have conserved segments at both ends, unlike the other orders, which typically have N-terminal prefixes and C-terminal suffixes that vary widely in length and composition. Among the 455 complete (H)KnS Dhns, sequences with a single copy of each component segment (HKS) are most common, at about 62% of the total, while an additional 6% have two or three K segments, 23% lack the terminal S segment, and only 8% lack well-defined H segments. Other variations on this consensus structure, such as multiple or internal, H segments are rare.

The generalized structure for F(S)Kn Dhns is as follows:

• An N-terminal phi segment comprising usually 10 to about 30 residues, highly variable in sequence but generally rich in Gly and polar amino acids.
• A single F segment.
• A second variable phi segment.
• A single S segment of 11–13 residues.
• An S-K transition segment composed mainly of Gly, Glu, and Lys (Figure 5), 8–30 residues in length.
• Usually 2 or 3 but as many as 13 K segments separated by 10–50 residue phi segments.
• A 10–30-residue C-terminal phi segment.

Typical Yn(S)Kn Dhns have the following structure:

• A 4–16-residue N-terminal phi segment, highly variable in composition.
• Typically 1–3 but as many as 36 Y segments, separated by 2 residues, with the first charged and the second variable.
• A highly variable phi segment.
• An 11–15-residue S segment, usually with a His-Arg or Arg-Arg prefix and ending with Glu-Asp.
• A conserved S-K transition segment adhering to the consensus sequence DGXGGR.
• Most commonly two but from one to as many as twelve K segments, separated by highly variable phi segments.
• A 2–7-residue C-terminal phi segment with 1–4 His residues.

These characteristics are generally consistent across the full phylogenetic breadth of at least angiosperms and in some cases earlier land plant groups, and so they have been conserved over at least 125 million years of land plant evolution.

This clear delineation of Dhn phylogeny and structural grouping should provide a strong foundation for future research on Dhn diversity, evolution, and function. One possible avenue would be localization studies to determine if the three main structural types are generally associated with specific cellular compartments or membrane systems as suggested in a review of extreme low temperature tolerance in woody plants [28]. This might guide further research on the role of accessory segments in modulating membrane binding or other behaviors specific to the different cell compartments.

### 4.4. Conserved Structure Implies Conserved Function

In intrinsically disordered proteins, such as dehydrins, conserved segments or domains likely impart conserved functions to the protein. Functional studies of the five conserved segment types are uncommon in the Dhn literature, with most being focused on the K segment as a functional unit, with the most incisive of these using deletion or substitution to investigate the role of intact or modified variants of the segment in membrane binding, cryoprotection, or other properties.

### 4.5. K Segments Bind Membranes and Cryoprotect Proteins

In a pair of studies on a maize K2 Dhn, Koag et al. demonstrated that the protein binds anionic phospholipid vesicles, accompanied by a gain in helicity attributed to the K segment, which adopts an amphipathic α-helix structure in nonpolar environments [29]. Both the native protein and a modified version with one K segment deleted bind to liposomes containing anionic phospholipids, while no binding occurred either in proteins modified by deletion of both segments or in lipids lacking anionic phospholipids [10]. Similarly, a *Vitis* K2 Dhn binds acidic liposomes via K segment Lys residues and prevents freezing-induced fusion [30]. This suggests that membrane binding is a primary function of the K segment.

Studies of Lti30, a K6 Arabidopsis Dhn with paired His residues flanking the K segments, confirmed binding to membranes with a negative surface potential, with K segments adopting an α-helical structure [31] and showed that phosphorylation of Thr residues and reversible protonation of the flanking His residues regulate binding behavior [11]. Further work with Lti30 demonstrated that the protein stabilizes multilamellar membrane systems via the electrostatic bonding of positively charged Lys and protonated His residues due to negative charges on phospholipid headgroups, thereby preventing the transition to non-lamellar structures and preserving membrane integrity [32].

The K segment may also be involved in binding and protecting proteins. Both native Dhns and isolated K segments prevent freezing-induced aggregation, inactivation, and denaturation of lactate dehydrogenase (LDH), an enzyme that is commonly used to assess cryoprotective activity [33]. In the same study, experimental replacement of hydrophobic residues in isolated K segments with Thr, Lys, or Glu abolished cryoprotective activity, while the replacement of charged residues with Thr had little effect. This supports a transient hydrophobic interaction model, where the hydrophobic residues in the K segment bind or interact with the hydrophobic regions of the enzyme to prevent freeze-dehydration-induced damage [33].

### 4.6. F and H Segments Are Also Implicated in Protein Binding and Protection

F segments isolated from four different Arabidopsis Dhns had similar levels of cryoprotective activity as the typical K segment [34]. Substituting the hydrophobic residues at the core and C terminal end of the segment with Lys, Glu, or Thr abolished cryoprotection, while activity was maintained when polar residues were replaced by Thr. An isolated H segment from an Arabidopsis HKS Dhn also showed cryoprotective activity in the LDH assay [35]. There seem to have been no other functional studies of these two more recently described conserved segments, notably with respect to membrane binding.

### 4.7. Closely Spaced Y Segments May Be Functional Units

Since the Y segment was first described as a common element in Dhn architecture nearly 30 years ago [3], there have been some suggestions as to its possible function [9] but few studies of its function. The observation reported here that Y segments usually occur in closely spaced multiples suggests that these poly-Y “supersegments” would be an appropriate subject for future functional studies. One study employing experimental modification of a *Physcomitrella* Dhn PpDHNA (NCBI accession XP_024395993.1) suggests a functional role for multiple Y segments. The native protein comprises a single C-terminal K segment preceded by 11 repeats incorporating a 6-residue version of the Y segment. Reducing the number of Y segments impaired protection of LDH and survival of *E. coli* cells under heat stress, as well as survival and growth performance of transformed tobacco plants under combined heat and dehydration stress, while deletion of only the K segment had a more limited impact [36].

### 4.8. S-K and K-S Segments Are Likely Functional Units

S segments can be phosphorylated, thereby affecting the binding and localization behavior of the protein [9]. Nuclear localization of a GFP-labeled FSK3 Dhn from *Triticum* (NCBI ABV24865.1) expressed in *Nicotiana* leaf cells was not affected by deletion of the three K segments, but with deletion of the S segment, the protein was dispersed in both the nucleus and cytoplasm, indicating that the S segment is required for nuclear localization [37]. The authors did not recognize the F segment, and both it and the S-K linkage were not modified in the deletion experiments, so it is unclear what role these parts of the protein may play in nuclear localization.

The conserved DGXGGR linkage in Yn(S)Kn Dhns was described only recently [15] and the more extended variants in other Dhn orders are first described here, so there have been no functional studies of linked S-K and K-S domains to date. The conserved mix of multiple Gly and charged residues in all types of S-K and K-S linkages suggest that the flexibility imparted by Gly and the charge or hydrophilicity imparted by negatively charged Asp and Glu and positively charged Lys and Arg are necessary features that allow the more conserved S and K segments to function in tandem. Furthermore, the relatively high abundance of His residues in (H)KnS K-S linkages may also contribute to pH-dependent modulation of membrane binding behavior, as discussed above.

### 4.9. Repeat Structures Contribute to Dehydrin Diversification

Tandem repeats in phi segments were noted as a general property in the early description and definition of the Dhn structure [4] but seem to have received little attention since then. Tandem repeats are a common feature of intrinsically disordered proteins and evolution by repeat expansion likely contributes to functional diversification [16]. Similarly, the diversity of repeat structures documented here suggests that repeat expansion is a common mode of evolution in Dhns and could lead to functional diversification, including by proliferation of the functional segment types. Functional studies have focused on Dhns with up to six K segments or similarly small numbers of other segments, so it remains to be seen if “more is better”, that is, if higher numbers of functional segments or the expansion of phi segments improve the protective properties of Dhns.

## 5. Main Conclusions

The Dhn Decoder script is a useful tool for identifying Dhns and Dhn-like proteins in input sequences, parsing these sequences to construct a structural formula, and for the preliminary identification and parsing of repeat structures. The results can be used to explore variations in Dhn structure, including sequence variation in the five conserved segment types, lesser-conserved variation in the regions between conserved segments, and repeat structures. The script and the Dhn database should be of interest to anyone interested in exploring Dhn diversity, evolution, and function in the full range of land plant species, including many ecotypes, and in well-documented species and varieties of agricultural and economic importance.

## Figures and Tables

**Table 1:** Distribution of Dhn types among phylogenetic groups for 2658 complete Dhn sequences identified by Dhn Decoder.

Dhn Group or Order							
Taxon	Kn	SKn	HKn(S)	F(S)Kn	Yn(S)Kn	Irr	Total
Algae	5	2					7
Bryophyte	18				5	4	27
Lycophyte		3	2				5
Fern	3		6				9
Gymnosperm	26	8		36		3	73
Basal angiosperm	1	3	1	1	2		8
Magnoliid		2	2	6	7		17
Monocot	92	91	54	45	232	16	530
Basal eudicot	13	6	17	24	26	1	87
Caryophyllid	3			22	16	1	42
Rosid	127	40	266	302	448	23	1206
Asterid	76	75	107	154	228	7	647
Grand total	364	230	455	590	964	55	2658

## Data Availability

All data are provided in Supplementary Materials.

## Supplementary Materials

The following supporting information can be downloaded at: https://www.mdpi.com/article/10.3390/biom15010137/s1, File S1: Dhn Decoder R script and input data (zip file). File S2: Dhns in reference genomes. File S3: Dhn Database (Excel file). File S4: Examples of Dhns with repeat structures. File S5: Supplementary tables and figures.
