# Supplementary material for: Order in Chaos: Lesser-Conserved and Repeat Structures in Dehydrins
Source: Biomolecules. 2025 Jan 16;15(1):137. doi: 10.3390/biom15010137 (PMC11763121; doi:10.3390/biom15010137)
Supplement: Supplementary file 1 [file biomolecules-15-00137-s001.zip › S2 Dhns in reference genomes.pdf]

Supplementary File S2. FASTA sequences for land plant Dhns identified by Dhn Decoder referenced to known Dhns in the Phytozome genome database.

FASTA sequences for the 17 Arabidopsis Dhn sequences identified by Dhn Decoder, referenced to the 10 Dhns in the TAIR genome

TAIR AT1G54410 HIRD11 HSK
```
>NP_175843.1 dehydrin family protein [Arabidopsis thaliana]
MAGLINKIGDALHIGGGNKEGEHKKEEEHKKHVDEHKSGEHKEGIVDKIKDKIHGGEGKSHDGEGKSHDG
EKKKKKDKKEKKHHDDGHHSSSSDSDSD
```

TAIR AT4G38410 GC-type FSKn
```
>NP_195554.2 Dehydrin family protein [Arabidopsis thaliana]
MADHPRSTEQQEADAAASKGCGMFDFLKKKPEDVHSSENARVTKEPKEEEKPSLAERFHLSDSSSSDEEA
GENGEKKEKKKKKKNEVAEDQCETEEKIPAGIGHEDGKEKGFMEKIKDKLPGGHNGKPEAEPHNDKAKE
KGFMEKIKEKLPGHTNDEKKKET
>CAD5330306.1 unnamed protein product [Arabidopsis thaliana]
MADHPRSTEQQEADAAASKGCGMFDFLKKKPEDVHSSENARVTKEPKEEEKPSLAERLHLSDSSSSDEEA
GENGEKKEKKKKKNEVAEDQCETEEKIPAGIGHEDGKEKGFMEKIKDKLPGGPDIYSITNDNLIINVHN
DLDEPFLLSWNGVQLRKNSYQDGVYGTTCPIPPGKNYTYAIQVKDQIGSFFYFPSLAVHKAAGGFGGFRI
LSRPRIPVPFPEPAGDFTFLIGDWFKHDHKVLKAILDRGHKLPLPQGVLINGQGVSYMSSITVHKGKTYR
FRISNVGLQNTLNFRIQGHQMKLVEVEGTHTVQSMYTSLDIHVGQSYSVLVTMDQPDQDYDIVVSTKFVA
KKLLISSTIHYSNSRYSHSSSANSVLVQQPADELDWSIKQARSIRTNLTASGPRPNPQGSYHYGRIKISR
TLILESSAALVKRKQRYAINGVSFVPGDTPLKLADYFKIKGVFKMGSIPDKPRRGRGMRMETSVMGAHHR
DFLEIIFQNREKIVQSYHLDGYSFWVVGMDRGTWSKASRREYNLRDAISRSTTQVYPESWTAVYVALDNV
GMWNLRSEYWARQYLGQQFYLRVYSPTHSLRDEYLLPKNALLCGRASNKHTTP
```

TAIR AT1G20450 ERD10/LTI45 DR-type FSK3
```
>NP_564114.2 Dehydrin family protein [Arabidopsis thaliana]
MAEEYKNTVPEQETPKVATEESSAPEIKERGMFDFLKKKEEVKPQETTTLASEFEHKTQISEPESFVAKH
EEEEHKPTLLEQLHQKHEEEEENKPSLLDKLHRSNSSSSSVSKKGEDGEKKKKEKKKKIVEGDHVKTVEE
ENQGVMDRIKEKFPLGEKPGGDDVPVVTTMPAPHSVEDHKPEEEEKKGFMDKIKEKLPGHSKKPEDSQVV
NTTPLVETATPIADIPEEKKGFMDKIKEKLPGYHAKTTGEEEKKEKVSD
>AAN60289.1 unknown, partial [Arabidopsis thaliana]
MAEEYKNTVPEHETPKVATEESSAPEIKERGMFDFLKKKEEVKPQETTTLASEFEHKTQISEPESFVAKH
EEEEHKPTLLEQLHQKHEEEEENKPSLLDKLHRSNSSSSSSSDEEGEDGEKKKKEKKKKIVEGDHVKTVE
EENQGVMDRIKEKFPLGEKPGGDDVPVVTTMPAPHSVEDHKPE
>AAK82471.1 At1g20450/F5M15_20 [Arabidopsis thaliana] (partial)
MDRIKEKFPLGEKPGGDDVPVVTTMPAPHSVEDHKPEEEEKKGFMDKIKEKLPGHSKKPEDSQVVNTTPL
VETATPIADIPEEKKGFMDKIKEKLPGYHAKTTGEEEKKEKVSD
>CAA54705.1 lti45, partial [Arabidopsis thaliana]
GKTQISEPESFVAKHEEEEHKPTLLEQLHQKHEEEEENKPSLLDKLHRSNSSSSSSSDEEGEDGEKKKKE
KKKKIVEGDHVKTVEEENEGVMDRIKEKFPLGEKPGGDDVPVVTTMPAPHSVEDHKPEEEEKKGFMDKIK
EKLPGHSKKPEDSQVVNTTPLVETATPIADIPEEKKGFMDKIKEKLPGYHAKTTGEEEKKEKVSD
```

TAIR AT1G76180 DR-type FSK2
```
>NP_001185408.1 Dehydrin family protein [Arabidopsis thaliana]
MAEEIKNVPEQEVPKVATEESSAEVTDRGLFDFLGKKKDETKPEETPIASEFEQKVHISEPEPEVKHESL
LEKLHRSDSSSSSSSEEEGSDGEKRKKKKEKKKPTTEVEVKEEEKKGFMEKLKEKLPGHKKPEDGSAVAA
APVVVPPPVEEAHPVEKKGILEKIKEKLPGYHPKTTVEEEKKDKE
```

TAIR AT1G20440 COR 47 DR-type FSKn
```
>BAH56867.1 AT1G20440 [Arabidopsis thaliana]
MAEEYKNNVPEHETPTVATEESPATTTEVTDRGLFDFLGKKEEEVKPQETTTLESEFDHKAQISEPELAA
EHEEVKENKITLLEELQEKTEEDEENKPSVIEKLHRSNSSSSSSSDEEGEEKKEKKKKIVEGEEDKKGLV
EHPEEKKGILEKIKEKLPGYHAKTTEEEVKKEKESDD
>NP_173468.1 cold-regulated 47 [Arabidopsis thaliana]
MAEEYKNNVPEHETPTVATEESPATTTEVTDRGLFDFLGKKEEEVKPQETTTLESEFDHKAQISEPELAA
EHEEVKENKITLLEELQEKTEEDEENKPSVIEKLHRSNSSSSSSSDEEGEEKKEKKKKIVEGEEDKKGLV
EKIKEKLPGHHDKTAEDDVPVSTTIPVPVSESVVEHDHPEEEKKGLVEKIKEKLPGHHDEKAEDSPAVTS
TPLVVTEHPVEPTTELPVEHPEEKKGILEKIKEKLPGYHAKTTEEEVKKEKESDD
```

TAIR AT3G50970 LTI30/XERO2 K6

>NP_190666.1 dehydrin family protein [Arabidopsis thaliana]
MNSHQNQTGVQKKGITEKIMEKLPGHHGPTNTGVVHHEKKGMTEKVMEQLPGHHGATGTGGVHHEKKGMT
EKVMEQLPGHHGSHQTGTNTTYGTTNTGGVHHEKKSVTEKVMEKLPGHHGSHQTGTNTAYGTNTNVVHHE
KKGIAEKIKEQLPGHHGTHKTGTTTSYGNTGVVHHENKSTMDKIKEKLPGGHH
>AAN60327.1 unknown, partial [Arabidopsis thaliana]
MNSHQNQTGVQKKGITEKIMEKLPGHHGPTNTGVVHHEKKGMTEKVMEQLPGHHGATGTGGVHHEKKGMT
EKVMEQLPGHHGSHQTGTNTTYGTTNTGGVHHEKKSVTEKVMEKLPGHHGSHQTGTNTAYGTNTNVVHHE
KKGIAEKIKEQLPGHHGTH

TAIR AT4G39130 Y2K

>NP_195624.1 Dehydrin family protein [Arabidopsis thaliana]
MADLKDERGNPIYLTDAHGEPAQLMDEFGNAMHLTGVATTVPHLKESSYTGPHPITAPVTTTNTPHHAQP
ISVSHDPLQDHDLRWFGTSSTEENGEGVGRKTNITDETKSKLGVDKPSAATVTGSGSGSVHEKKGFFKKI
KEKLSGHHNDL

TAIR AT3G50980 XERO1 YSK2

>NP_190667.2 dehydrin xero 1 [Arabidopsis thaliana]
MESYQNQSGAQQTHQQLDQFGNPFPATTGAYGTAGGAPAVAEGGGLSGMLHRSGSSSSSSSEDDGLGGRR
RKKKGITEKIKEKLPGHHDSNKTSSLGSTTTAYDTGTVHHEKKGMMEKIKEKLPGGHH
>CAD5325456.1 unnamed protein product [Arabidopsis thaliana]
MESYQNQSGAQQTHQQLDQFGNPFPATTGAYGTAGGAPAVAEGGGLSGMLHRSGSSTTTAYDTGTVHHEK
KGMMEKIKEKLPGMVKSMVCIVLAQFSLVALFPLCICYQTHQSTSSVASLSPQFYDNSCPNAQAIVQSYV
ANAYLNDPRMAASILRLHFHDCFVNGCDASVLLDSSGTMESEKRSNANRDSARGFEVIDEIKSALENECP
ETVSCADLLALVARDSIVICGGPSWEVYLGRRDAREASLIGSMENIPSPESTLQTILSMFNFQGLDLTDL
VALLGSHTIGNSRCIGFRQRLYNHTGNNDPDQTLNQDYASMLQQGCPISGNDQNLFNLDYVTPTKFDNYY
YKNLVNFRGLLSSDEILFTQSIETMEMVKFYAENEGAFFEQFAKSMVKMGNISPLTGTDGEIRRICRRVN
HDV

TAIR AT2G21490 Y2SK2

>NP_179744.1 dehydrin LEA [Arabidopsis thaliana] (AT2G21490)
MADLRDEKGNPIHLTDTQGNPIVDLTDEHGNPMYLTGVVSSTPQHKESTTSDIAEHPTSTVGETHPAAAP
AGAGAATAATATGVSAGTGATTTGQQHHGSLEEHLRRSGSSSSSSSEDDGQGGRRKKSIKEKIKEKFGSG
KHKDEQTPATATTTGPATTDQPHEKKGILEKIKDKLPGHHNHNHP

TAIR AT5G66400 RAB18/ATD18 Y2SK2

>NP_201441.1 Dehydrin family protein [Arabidopsis thaliana]
MASYQNRPGGQATDEYGNPIQQQYDEYGNPMGGGGYGTGGGGGATGGQGYGTGGQGYGSGGQGYGTGGQG
YGTGTGTEGFGTGGGARHHGQEQLHKESGGGLGGMLHRSGSGSSSSSEDDGQGGRRKKGITQKIKEKLPG
HHDQSGQAQAMGGMGSGYDAGGYGGEHHEKKGMMDKIKEKLPGGGR

FASTA sequences for homologues identified by Dhn decoder to the eight Dhn genes in the *Oryza sativa* genome on Phytozome.

Os11g26570 YSK2
>XP_052136541.1 water stress-inducible protein Rab21-like [Oryza glaberrima]
MAHFQGQQHGHPAARVDEYGNPVAAGHGITGTQEAGAGGYGAAGNVAQPQHAFGGATDAGGYGRQAGYGA
TGTGTHDAAGYGGSGQPAYGATGTGVHDAGGLMPGHTAGHGTTGTGVHHGAGAGLPAEQTAGYETTGVTG
AQHGAGGLGTGHTAGYGTTGAHHGAGGLGTGHMAGHGATTTPDTMAYGTTGTGAPHGATAGTGAYPHAGG
QFQPAREEHKTGGILRRSGSSSSSSSSEDDGMGGRRKKGIKEKIKEKLPGGNKGGQQQPTATAATGGYGA
GTGHTAAAGTTTDAGGTAYTPTTQPTHEKKGMMEKIKEKLPGGGHH

Os11g26750 YSK2
>NP_001410121.1 dehydrin Rab16D [Oryza sativa Japonica Group]
MEYQGQHGGHASSRADEHGNPAVTTGNAPTGMGAGHIQEPAREDKKTDGVLRRSGSSSSSSSSEDDGMGG
RRKKGIKEKIKEKLPGGNKGNNQQQQQEHTTTTTGGAYGPQGHDTKIATGAHGGTAATTADAGGEKKGIV
DKIKEKLPGQH

Os11g26760 YSK2YSK2
>XP_006662923.2 cold shock protein CS66-like [Oryza brachyantha]
MEHQGQHGHVTSRVDEYGNPVGAGAGHGQMGTGGMGTHGTTGGMGTHGTTGTGTGGQFQPMREEHKTGGV
LQRSGSSSSSSSEDDGMGGRRKKGIKEKIKEKLPGGNKGQQHTMGGTGTGAGAYGQQGHGTGITGTTGTT
GAHGTTTTTETGEKKGIMDKIKEKLPGQEREEMENYQGQHGYTADRADVYGNPVYDGAPGGGPAVGMGAA
GHGAGGGQFQPAREGRKTGGILHRSGSSSSSSSSEDDGMGGRRKKGIKEKIKEKLPGGNKGTQQQQMMDG
DTGGYGAYGQQGHAGMTGGGTGLHGAEYGTTGEKKGFMDKIKEKLPGQH

Os11g26780 YSK2
>NP_001410123.1 dehydrin Rab16B [Oryza sativa Japonica Group]
MDNYQGQHGYGADRVDVYGNPVGAGQYGGGATAPGGGHGAMGMGGHAGAGAGGQFQPAREDHKTGGILHR
SGSSSSSSSSEDDGMGGRRKKGIKEKIKEKLPGGNKGNNHQQQQMMGNTGGAYGQQGHAGMTGAGTGVHG
AEYGNAGEKKGFMDKIKEKLPGQH

Os11g26790 YSK2
>NP_001410124.1 water stress-inducible protein Rab21 [Oryza sativa Japonica Group]
MEHQGQHGHVTSRVDEYGNPVGTGAGHGQMGTAGMGTHGTTGGMGTHGTTGGMGTHGTTGTGGGQFQPMR
EEHKTGGVLQRSGSSSSSSSEDDGMGGRRKKGIKEKIKEKLPGGNKGEQQHAMGGTGGAYGQQGHGTGMT
TGTTGAHGTTTTDTGEKKGIMDKIKEKLPGQH

Os02g44870 FSK3
>KAF0934351.1 hypothetical protein E2562_024837 [Oryza meyeriana var. granulata]
MEDERNTQSHQGGEATEQVEVKDRGLFDSLLGKKKEDQPEKKKQEEELVTGMQKVSVEEPKKEEHHAEGE
KKESLLSKLHRSSSSSSSSSDEEEEVIDDNGEVVKRKKKKGLKEKIKEKLPGHKNHDGEPAPPPVATGFP
APAPPAPVVAAAPTPAPAPVVTHGGHHDTAVPVEKIEGDHAKTDASHAPEEEKKGFLDKIKEKLPGGHKK
PEDATAAPPAAAPAAPSPAPAPAHPPPATEEVSSPDGKEKKGILGKIMEKLPGYHKGSGEEDKTAATTAG
EHKSTA

Os01g50700 Y2SK2
>EEE55258.1 hypothetical protein OsJ_03161 [Oryza sativa Japonica Group]
MVVDSAYYDVLGVSTDASAAEIKKAYYLKAKLVHPDKNPNNPDAERRFKELGEAYQILSDPVRKDSYDKH
GKEGLPQDNMIDPTAVFGMLFGSDYFEDYVGQFALASVASVEIEEESDNTEARARIQDKIKELQTEREQK
LVQSLKDRLQPYVDGMQDEFGDWAGAEAQRLSQAAFGEAMLHTIGYIYARQAARELGKSKMYMGVPFIAE
WVRDKGHHVKSQVNAAAGAISLIQLQEGIKKIEGDDKEGQLMKSIEEKKDAMLNSLWKINVVDIESTLSR
VCQAVLRENTVSKDVLKVRARGLKKLGTIFQCTLLLFTRNLPEIRPQRGAPGAYVPFSGHARRPYEGTRG
LPAGLQIPPVASCFLVQIRVRFRFIIIQFPISSIARITHGSKRVTELETEEEATRRHQPMAEHATGVYGH
PYPRVDQYGNPVPPVDQYGNPVPDEPAPRDTAAGYVAPPDPAVSTGDYGLAGAEAPHPHESAVMSGAAAA
AVAPGGEAYTRDGGGVVPPAGEKTFAYEGTVSAAGVTGASGQLQPTTREEGHTTLGETLRRSGKSSSSSS
SSSEDDGQGGRRKKKSIKEKIKEKLPGSHKQEEQKQAGHTAPAAGTGTGTGTHAAGKHEKKGIVEKIKEK
LPGHGHH

Os03g45280 HKS

>BAA24979.1 LIP5 [Oryza sativa Japonica Group]
MAGIIHKIEEKLHMGGGEHKKEDEHKKEGEHHKKDGEHKEGVVEKIKDKITGDHGDGGEHKEKKDKKKKK
EKKHGEEGYHHDGHSSSSSDSD

FASTA sequences for the two Dhn genes identified by Dhn Decoder in *Amborella trichopoda*, referenced to the equivalent Phytozome identifier, and a third HKnS Dhn not identified as a Dhn in Phytozome.

evm_27.TU.AmTr_v1.0_scaffold00001.295 SK3
>XP_006829002.1 dehydrin COR47 [Amborella trichopoda]
MADEHTTSVVPESGDGRVEVHERGLFGHHHPETPEQKQEEALASKFEEVYVSEEKKEEKEKDEEKKGSLL
EKLHRSGSSSSSSSSDEEEAEEWKKKKKERKGLKEKIKEKLSGGSEAVKEKAGEGAPHCAGAEHAHEKEK
KGLPEKIKEKLHGGGGYGAPQHPVEAPESAPQTSAPHPEGQSHEEKKGFLEKIKEKFSGYHPNSGEETTP
SH

evm_27.TU.AmTr_v1.0_scaffold00082.27 SK2
>XP_006833478.1 dehydrin DHN2 [Amborella trichopoda]
MAEYRGDERYQKHEHGYEQGQPGYYGGTVTGTGTGAGTGAGTGTGGVMLGTTGAGMMHGTQPEQQKLHRS
NSSSSSSSEDDGMGGRRKKKGLKEKIKEKLPGGHKTTEGQGHEAGAGVHGHEHEHEKKGIIEKIKEKLPG
HHKDQLNA

Not identified as a Dhn in Phytozome:
evm_27.model.AmTr_v1.0_scaffold00004.97 HKnS
>XP_006826344.1 protein SRC1 [Amborella trichopoda]
MASIVNKIGEKLGIGGDKKHGEEHKHGEEHKHGEELKHHKEGEHSHGEHGEHKEGFMEKVKEKIHGDGDK
IHGDGEKEKKKKKKKKKKPGHEHDGGSSSSSSDSD

FASTA sequences for two (H)KnS Dhns identified by Dhn Decoder in *Selaginella moellendorffii*, not previously identified as Dhns in Phytozome.

S. moellendorffii genome ID in Phytozome: 446994 HKS
>XP_002987557.1 uncharacterized protein LOC9629804 [Selaginella moellendorffii]
MASMMEKISDKLHMGHKKDQVAHNTAPSHATPVQSSTPPSHNTAPGSYGTPQPGMATTTTSTTTTQPQKE
GLMDKIKKKKNQHKEKKKAGGGSSSSSSDSD
>XP_024522267.1 uncharacterized protein LOC112343298 [Selaginella moellendorffii]
MFIHFPSFQLEAKIPSLQISHKAMASMMEKISDKLHMGHKKDQVAHNTATPVQSSTPPSHNTAPGSYGTP
QPGMATTTTSTTTTQPQKEGLMDKIKKKKNQHKEKKKAGGGSSSSSSSSDSD

Not identified as a Dhn in Phytozome:
448256 KS
>EFJ07973.1 hypothetical protein SELMODRAFT_448256 [Selaginella moellendorffii]
MAFRSVLVASKKNALGHLLVKKEFLATSPLLLNKEAGTAESMAHKVGDKVEKAGENLKETAKDSKEPAES
GMREQVRSAAEKIGDATASAGDKIEHSMESEGGGEEKSKLNLEDKQIFGFVLFSRSQVLFAVLLILAALM
ASMMEKIGEKFGKKDDEHNKLGGQTHETHGLGQQTHGQGAGYGQEAGYGQHGQGVGHGQGVGHNPGSTPY
GATTGTTGTGHNPGSTPYGATTGTNPTSGHTATGQPQKKEGLMDKIKKKKNQHKERKEGDKSSSSSESD

FASTA sequences for the five Dhns in Phtyozome identified by Dhn Decoder in *Aquilegia coerulea,* and two additional sequences not previously identified as Dhns in Phytozome.

Aqcoe6G128700 Y2SK2
>PIA52794.1 hypothetical protein AQUCO_01000572v1 [Aquilegia coerulea]
MSGYPIQSTDEHGNLVPQRDEYGNLVHQTGTGHTGTGQTGPGTGHTGTGGYGGHTGTGGYGAGDGTGTGQ
HTGTGLGTGTGQHAGTGLGTGAGHHTGTGVGGEQHGGVLRRSGSSSSSSSSEDDGMGGRRKKGLKQKIKE
KLPGGQKETQPGYGAGTTGAGTTGPGYGTTTGAGAGYGTTEQPHEKKGMIEKIKEKLPGHHA

Aqcoe6G197600 FSK3
>PIA32914.1 hypothetical protein AQUCO_04300099v1 [Aquilegia coerulea]
MAEQQPQTTVEGAEIKDRGMFDFLSKKKEEEKPQEAEVLATELENVHVSEPVHDHHVAEPVEVKKEEVDE
EKKPSLLEKLHRSDSSSSSSSSEEEVEEDGQKIKRKKNKGLKEKIKEKISPEEKKVEEDTTVPIEKFDDE
VGVVSHEGEVKHPEEKKTFMEKIKEKLPGGNKKTADHESASTPDAIVLPPPAAPVHEEEPKEKKGILEKI
KEKLPGYHKTEDEKKETTTTTEH

Aqcoe5G186200 YK3
>PIA55858.1 hypothetical protein AQUCO_00700288v1 [Aquilegia coerulea]
MADQQDVWGTARNTDEFGNPRAPKRTDDDETLATGGMGHQQLHRGRDEHDKNMMDKVKEKVPGSHTGTTD
TGAGYGTGQKTAGCGHDEKGMMDKINEKMPGSHTSETGGCGTGQKQTGFGAGQQTGYGREHEDKGMMEKV
KEKLPGTGRHQP

Aqcoe5G125000 SK4
>PIA56789.1 hypothetical protein AQUCO_00700853v1 [Aquilegia coerulea]
MAAEQIQHPLTNESNVEEHETNDRGILNFLEKKEEENKPDDELSVPEFAETHVLKSEEKKQEEENKPSLT
EKLHRSNSSSSSSDEEGDEGGKKKSKGLKDKIKEKIGGEKEQEKNAEHNQVVAGDVTRPEGAEDKKGFAE
KIKEKLPGTHKTDNNEELVSPDITHQEEKEEKKGFMEKIKEKKLPGTNKTDEVAPVDKYHPTEANKHQDE
SEEKKGFMEKIKEKLPGIPANQKNVEVKGEKETGNYY

Aqcoe5G125200 K2
>PIA56786.1 hypothetical protein AQUCO_00700851v1 [Aquilegia coerulea]
MATEQNQIPKAHVTNEEEGQHEHEHHTMLDFLEKKEEQKPTDELSVNEFVKDHVSKSEEKKEEEEEHKPT
SAPNVDRSSDIHSSGDVTHPEETEEKNLMEKIKNKLPGAHKKTDEVAPVEHHPTETKTNIHQDKPEEKKG
LMEKIKEKLPGHNKSVEEEKGAI

Not identified as Dhns in Phytozome:
Aqcoe2G065100.1.p HKS
>PIA59837.1 hypothetical protein AQUCO_00400603v1 [Aquilegia coerulea]
MAGIMNKIGDALHIGGNKEEQKQGEAHKTEIKHGETAHKPEQKEGFVDKIKDKITGQGPHKTEGGGEKKK
KKEKKKKEGDHGHDGSSSSSDSD

Aqcoe1G498400.1.p K1
>PIA46155.1 hypothetical protein AQUCO_01600430v1 [Aquilegia coerulea]
MPMKILDVPCLEDNYSYIIFDESSKQAAVVDPVEPDKILQVVNQHGLHLNLVLTTHHHWDHAGGNDKIKE
LLPGIKVYGGSLDNVRGCTHQLLNGDTLSLPHTNINILSLHTPCHTRGHISYYVTATDQHDSAVFTGDTL
FVAGCGKFFEGTAEQMYQSLCVTLGSLPKTTRVYCGHERMRR
