# Supplementary material for: Order in Chaos: Lesser-Conserved and Repeat Structures in Dehydrins
Source: Biomolecules. 2025 Jan 16;15(1):137. doi: 10.3390/biom15010137 (PMC11763121; doi:10.3390/biom15010137)
Supplement: Supplementary file 1 [file biomolecules-15-00137-s001.zip › S4 Example sequences.pdf]

Supplementary File S4: Examples of sequences with extended or complex repeat structures, with accession ID and name from NCBI.
Color coding: brown: H segment; blue: F segment; green: S segment; purple: conserved Y segment; pink: low-scoring Y-segment inferred by position; red: conserved K-segment; orange: low-scoring K-segment inferred by position.

XP_021833744.1 cold-shock protein CS120-like isoform X1 Prunus avium Y2K10

Seg ID	Length	Start	Sequence
Nterm 0.1	72	1	MAHFGSTPEPTNTDEFGNPVHHSTTGTGRKDEFGNPVQHGVADTGYGTGAGYATHTKPSVVEHHGVPAAFNS
Rpt 1.1	41	73	KDDQYSRDSQTTTGGYGGDGYTGGEKKGILGQVKDKLPGGN
Rpt 2.1	44	114	KDDQYSHDTQTTTGAYGGAGYTGDDTREKKGIIGQVKDKLPGGQ
Rpt 3.1	44	158	KDDQYCRDSHPTTGTYGGAGCTGGENQEKKGVIGQVKDKLPGGQ
Rpt 4.1	44	202	KDDQFCRDTHPTTGAYGGAGYTGGEHQEKKGVIGQVKDKLPGGQ
Rpt 5.1	44	246	KDEQYSHDTHPSTGAFGGGGYTGDDTREKKGGIGQVKDKLPGGQ
Rpt 6.1	44	290	KDDQYSHDTRKTTGAYGGAGDTGDDTREKKGGIGQVKDKLPGGQ
Rpt 7.1	44	334	KDDQYSHDTRKTTGAYGGAGDTGDDTREKKGGIGQVKDKLPGGQ
Rpt 8.1	81	378	KDDQYCHDTHPSTGAFGGGGYTGDDTREKKGVADKVREKLPGGQNVHPTTGPYGGAGATGGETRERRGVADKVKEKLPCGP
Rpt 9.1	50	459	KDDQYSHDTHPTNPTSAGHGGVGHTGGEPQLHEKKGLMEKIKDKLPGHNN

ADL59574.1 dehydrin 7 Populus alba x Populus glandulosa FK14

ID	Length	Start	Sequence
Nterm	80	1	MAGVNKSHEYETKTKAGGESGAAETRDRGLFGFMGKKKEEKPQEEVPATGYEENIHRSDNSYPGDGEKKHEHTTVPSNTE
Rpt 1	41	81	TPLEPEKKKSYFEQAKDMIPAYKKTEDAPPSPTEAAVHPTE
Rpt 2	40	122	TPLEPEKKSYFEQAKGMIPAYKKTEDGPPSPAETAVHPTE
Rpt 3	40	162	TPLEPEKKSYFEQAKGMIPAYKKTEDAPPSPTEAAVHPTE
Rpt 4.	41	202	TSLEPEKKKSYFEQAKERIPTFKKTEDAPSSPAKAAVHHTE
Rpt 5	29	243	TPLEPEEKRGFFDQAKERTPGFKKTEEVS
Rpt 6	29	272	PRREPEEKRGFFDQAKERTPGFKKTEEVS
Rpt 7	29	301	PRREPEEKRGFFDQAKERTPGFKKTEEVS
Rpt 8	29	330	PRREPEEKRGFFDQAKERTPGFKKTEEVS
Rpt 9	46	359	PRREPEEKRGFFDQAKERTPGFKKTEEVSPRPAKSAYNEGAFSQTG
Rpt 10	40	405	TPFEPEEKKGFLDKVKEKVPAHKTEEVPPPPAESAFSHTE
Rpt 11	40	445	TPFEPEEKKGLLEKVKEKVPSQKRTEEAPHPPAAAFSHTN
Rpt 12	42	485	TPFEPEEKRGFNKEKVPTHKKTGEFPFPAKPASTEAAVSNTN
Rpt 13	30	527	TPLEPEEKRGLLDKIKDKMPGHKKTDEVPP
Cterm	37	557	SEFDSTENVVSHKEEPVVKKGMMEKIKDKLPGHRPQI

AAL83426.1 60 kDa dehydrin-like protein *Cornus sericea* Y36SK2

ID	Length	Start	Sequence
Nterm	8	1	MAQYGNPT
Rpt 1	37	9	EGLYKQPTDVYGNPISRTHESGNPVQRTADAQYGNPT
Rpt 2	37	46	EGVYKQPTDVYGNSISRTHESGNPVQKTADAQYGNPT
Rpt 3	37	83	EGVYKQPTDAHGNPISRTHESGNPVQKTADAQYGNPT
Rpt 4	37	120	EGVYKQPTDAHGNPVSRTHESGNPVQKTADAQYGNPT
Rpt 5	37	157	EGVDKHPADAHGNPAFRTRESGNPVQKTADAQYGNPT
Rpt 6	37	194	EGVYKHPTDAHGNPVSRTHDFGNPVQKTADAQYGNPT
Rpt 7	37	231	EGVDKHPADAHGNTAFRTRESGNPVQKTADAQYGNPT
Rpt 8	37	268	EGVDKHPTDAHKNSISRTHDFGNPVQKTVDAQYGNPT
Rpt 9	37	305	EGVYKHPTDAHRNSISRTHNSGNPIQKTADAEYGNPT
Rpt 1	37	342	EGVHKHPTDAHENPLSRTHESDNPIQKTAGAQNRNPT
Rpt 11	37	379	EGVHKHPTDAHRNPVSRTHESGNPIQKTADAQYRNPT
Rpt 12	37	416	EGVHKHPTDAHGNPVSRTHEYGNPIRKIADGQGVHPT
Cterm1	47	453	GGTTEGYITTSGTSTGIGNGGAIGGQQHGGVLQRSGSGSSSSEDDGQ
Cterm2	67	500	GGRRKKKGLTDKKKEKLSGGRKPAQASHPTATTTTTTGHDIYKGGQQNQEKKGVMEKIKEKLPGQHN

KAE8057341.1 hypothetical protein FH972_014042 Carpinus fangiana F2K2

ID	Length	Start	Sequence
Nterm1	57	1	MAEHHHVKPSDESEAAPVGRDDHQGGVESKERGWFDFLGKKEDKKPQEEVLVSEFEN
Nterm2	58	58	DDHQGGVESKDRGWFDFLGKKEEKKPQEEVLVSEFENVSVSEPEPKVDQGYYKDEPKV
Rpt 1	11	116	EGYHKEEPKVD
Rpt 2	16	127	EGYHKEEPKVERPKVD
Rpt 3	21	143	QGYHKEEPKMEGYYKDELKVD
Rpt 4	10	164	QGYHEEEPKV
Rpt 5	11	174	EGYHKEEPKID
Rpt 6	10	185	DGYHKEEPKV
Rpt 7	11	195	EGYHKEEPKVD
Rpt 8	11	206	EGYHKEGPKVD
Rpt 9	10	217	QGYNKEEPKV
Rpt 10	21	227	EGYCKEEPKVKGYYKDELKVD
Rpt 11	10	248	QGYYKEEPKV
Rpt 12	11	258	GGYHKEEPKMD
Rpt 13	16	269	EGYHKEEPKVEGPKVD
Rpt 14	21	285	QGYHKEEPKVEGYYKDELKVD
Rpt 15	10	306	QGYHEEEPKV
Rpt 16	11	316	EGYHKEEPKID
Rpt 17	11	327	DGYHKEEPKVD
Rpt 18	16	338	EGYHKEEPKVEGPKVD
Rpt 19	10	354	QGYNKEEPKV
Rpt 20	21	364	EGYCKEEPKVKGYYKDELKVD
Rpt 21	10	385	QGYYKEEPKV
Rpt 22	11	395	GGYHKEEPKMD
Rpt 23	21	406	EGYHKEEPKVEGYYKDELKVD
Rpt 24	10	427	QGYHEEEPKV
Rpt 25	10	437	EGYYKEEPKV
Rpt 26	10	447	EGYHKEEPKV
Rpt 27	11	457	EGYHKEEPKVD
Rpt 28	11	468	EGYHKEEPKMD
Rpt 29	16	479	EGYHKEEPKVEGPKVD
Rpt 30	10	495	QGYHKEESKV
Rpt 31	21	505	EGYCKEEPKVEEYYKDELKVD
Rpt 32	10	526	QGYHEEEPKV
Rpt 33	20	536	EGYYKEEPKVASYYKEESKV
Rpt 34	10	556	EGYHKEEPKV
Rpt 35	10	566	AGYYKEEPKV
Cterm1	50	576	DESYKKEEERENKEMKEKKGHTLKEKIAGEKEEEKHEKYKEKHEKYEDTS
Cterm2	49	626	VPVHVEKYEEEVAHIPAEPAALPEEKKGFLGKIKEKLPGHHKKAEEVHS
Cterm3	50	675	PHPTSTEHASAALPPHYEGEASPKEKKGLLGKIKEKIPGYHPKTEEEKEK

KVI07719.1 Apolipoprotein A1/A4/E Cynara cardunculus var. scolymus K1

ID	Length	Start	Sequence
Nterm1	54	1	MAAIVFYWFCSCSCFAAAISSGLLCFLVLVAAIMADKSVCETVAVVKVEAEEDC
Nterm2	53	55	NEAVVLVEVDRKSGDDCVDGKTKKAELKEKIEEEKEKIGDKIHEAKYKVEEKA
Rpt1	11	108	EELKEKIECDV
Rpt2	18	119	EEAKEKIREKEYEHEYKK
Rpt3	18	137	EEHKEKLKEEVEEAKYKI
Rpt4	11	155	EEFKEKVETKE
Rpt5	11	166	GEIKEKIEEKI
Rpt6	18	177	EEFKEEIEEIVAEAKHKK
Rpt7	11	195	EELKEKIECEI
Rpt8	18	206	EEAKEKIKEKEIEHEYKK
Rpt9	18	224	EERKEKLKGEVDEAKYKI
Rpt10	11	242	EELEKKVECIE
Rpt11	11	253	EEVKEKIEEKI
Rpt12	18	264	EEFKEKIEKKVEEVKYEK
Rpt13	11	282	EELKEKIECEI
Rpt14	18	293	EEAKEKIKEKEYEHEYKK
Rpt15	11	311	EEHKEKLKEEV
Rpt16	18	322	EEAKYKIDEVKEKIECKE
Rpt17	11	340	EEVKEKIEEKI
Rpt18	18	351	EEIKEKIEEKVEEAKYEK
Rpt19	11	369	EELKEKIESEI
Rpt20	18	380	EEAKKEIKEREYEHEYKK
Rpt21	11	398	EEHKEKLKEEV
Rpt22	18	409	EEAKYKIEEFKENVEYKV
Rpt23	11	427	EEVKEKIEEKI
Rpt24	18	438	EEFKEKIEEIVAEGKHKK
Rpt25	11	456	EELKEKIKCEI
Rpt26	18	467	EEAKEKIKEKEYEHEYKK
Rpt27	18	485	EEHKEKRKEEVEVVKHKI
Rpt28	11	503	EELKEKVECIE
Rpt29	11	514	EEIKEKVEEKI
Rpt30	18	525	KEFKEKVEEKVEEVKYEK
Rpt31	11	543	EELKEKIECEI
Rpt32	18	554	EAAKEKIKEREYEHEHKK
Rpt33	18	572	EERKEKHKEEVEEAKHKI
Rpt34	11	590	EELKEKVECKE
Rpt35	11	601	EEVKEKIEEKI
Rpt36	11	612	EELKEKIECEE
Rpt37	18	623	EEIKEKIKEKEYEHEHKK
Rpt38	18	641	EERKEKHREEIAEAKHKI
Rpt39	11	659	EELKEKVECKE
Rpt40	18	670	EEVKEKIKELKEKIECEV
Rpt41	18	688	EEVKEKIKEKEIELEYKK
Rpt42	18	706	EEHEEKLKEEIEDVKHKI
Rpt43	11	724	EEIKENIECKV
Rpt44	18	735	EEIEEKIKEKIEEVKENI
Rpt45	11	753	EEKIEEHKEKK
Cterm1	56	764	ELKNEKKELEKEMKELEKIKKHEEERCEVVTLPEPSYEQEEKIVFVEKIEVKAEED
Cterm2	56	820	CVAAPPPPPPVAAHSDYTATVEVEHKEKKGIFEKIKQKLHGNHHSKSEEKKEKEHY
