# Supplementary material for: Order in Chaos: Lesser-Conserved and Repeat Structures in Dehydrins
Source: Biomolecules. 2025 Jan 16;15(1):137. doi: 10.3390/biom15010137 (PMC11763121; doi:10.3390/biom15010137)
Supplement: Supplementary file 1 [file biomolecules-15-00137-s001.zip › S5 Tables and figures.pdf]

Table S1. Percentage of K segments in Dhn groups with at least one or two His residues in the 6 positions N-terminal (prefix) or S-Terminal (suffix) to the segment.

K segment position	Kn	SKn	(H)KnS	F(S)Kn	Yn(S)Kn	All groups
**Prefix**						
**Nterm**						
At least 1	36.9	3.9	48.5	10.2	14.4	**16.5**
At least 2	6.1	0.4	11.7	1.5	2.6	**3.0**
**Mid**						
At least 1	59.2	31.3	14.7	35.8	63.6	**45.3**
At least 2	20.7	8.3	1.2	5.4	22.7	**12.9**
**Cterm**						
At least 1	43.4	43.7	74.0	48.0	72.4	**59.7**
At least 2	14.3	8.3	25.4	4.8	34.1	**20.5**
**All positions**						
At least 1	46.5	26.4	57.5	31.5	48.8	**42.5**
At least 2	9.6	2.1	3.0	2.2	8.9	**5.9**
**Suffix**						
**Nterm**						
At least 1	56.7	69.1	39.8	45.8	70.0	**59.8**
At least 2	22.4	18.0	2.9	6.9	10.7	**11.8**
**Mid**						
At least 1	67.0	90.6	14.1	61.8	57.1	**58.1**
At least 2	42.4	10.4	0.0	9.3	3.9	**13.9**
**Cterm**						
At least 1	58.7	81.6	48.0	88.9	88.6	**76.1**
At least 2	23.8	27.4	9.0	13.6	51.9	**29.5**
**All positions**						
At least 1	60.7	78.2	39.4	66.2	76.2	**66.8**
At least 2	29.0	21.1	6.2	10.0	28.1	**20.2**
**Prefix + Suffix**						
**Nterm**						
At least 1	69.2	69.1	68.0	49.2	75.1	**66.0**
At least 2	37.3	21.5	25.2	12.7	20.5	**21.0**
**Mid**						
At least 1	77.7	94.8	27.6	72.6	84.0	**73.0**
At least 2	60.2	35.4	2.5	30.2	53.5	**39.8**
**Cterm**						
At least 1	67.8	85.6	81.1	92.6	96.5	**87.2**
At least 2	42.3	49.1	54.5	52.7	73.2	**58.0**
**All positions**						
At least 1	71.1	80.7	67.6	71.9	86.3	**77.1**
At least 2	46.4	36.3	39.0	32.4	49.8	**41.9**

Table S2. Representative phi segments linking K and C-terminal S segments in (H)KnS Dhns. n is the number of segments of each length in the overall library of 374 (H)KnS Dhns identified by Dhn Decoder. In length classes with >2 sequences, 2-3 examples were randomly selected from all segments in that length class.

NCBI accession	Length	n	K-S phi segment sequence
XP_017252260.1	0	1	
XP_050910498.1	1	2	G
XP_017252260.1	1	2	L
KAI5064993.1	2	2	GE
KAJ0817778.1	2	2	HH
MQL74592.1	4	5	EEHH
XP_019054435.1	4	5	EHGG
XP_006826344.1	4	5	HEHD
XP_062209370.1	6	5	EGHHHG
MCO5555878.1	6	5	GKKGDA
MCO5607452.1	6	5	GKKGEA
ONK64184.1	7	6	AKCNSPD
RYR53894.1	7	6	EHGHDHG
XP_024522267.1	8	2	HKEKKKAG
XP_002987557.1	8	2	HKEKKKAG
KAG8097698.1	9	1	EGHHHDDGH
KAH9650592.1	10	2	KKEKKKHEDG
CAH1434251.1	16	1	DGGSKQQQQRQRLDHY
EPS66827.1	17	1	GEENKKKKKKDKKHDH
XP_058740102.1	18	3	EGEGGEKKKKERKKREDG
XP_028207705.1	19	5	EGEKKKKEKKKREHGHEDG

CAK8542876.1	19	5	GGEKKKKERKKREDGHEHG
XP_055815312.1	20	5	EDHKDGEEKKKKKKKEKKHD
KAB5512227.1	20	5	EGVKKKKEKKKKKEHEHGHD
KAG6506093.1	21	17	EEHGEKEGKKKKKKERKHDHD
AAL73185.1	21	17	EGHEEKKKKKKEKKHDEHGHD
XP_030926797.1	21	17	EGQGKPEEGKKKKKKEKKHDG
AEV59623.1	22	23	DGENGEGINKKKENKKKHEHGH
XP_027903653.1	22	23	EGGEGEKKKKKEKKKKHEDGHG
AEU08834.1	22	23	GEGGEKKKKKEKKKHGEGHEHG
MCE3215852.1	23	22	EDHKDGEEKKKKKKKKDKKEKND
XP_047975121.1	23	22	GDAEGADGKKKKKKKDKKKHGDD
CAI8584587.1	23	22	GEGGAGEKKKKERKKREDGHEHG
KAG5098211.1	24	47	DGHDKGEKKKKKDKKKKEHGHDHH
KAJ3685897.1	24	47	EEHGEKKDKKEKKKKKEKKEGHDG
XP_022719950.1	24	47	EGGEHGHDGEKKKKKKEKKKHEDG
AEV59617.1	25	41	DGESGEGIKKKKEKKKKHEHGHEHG
KAF3442207.1	25	41	EGGDHKGEKKKKKDKKKHEHGHDHG
NP_001351801.1	25	41	EGGEGENIKKKKDKKKRGEHGGEHG
CAK7341507.1	26	35	DGAHGEGEKKKKEKKEKKKKGEHGHD
MED6125970.1	26	35	GGAEGVDGEKKKKKDKKKGEHGKDHG
KAI3443383.1	26	35	GGGEQQGKEEKKKKKEKKKHEPGREH
AXS76868.1	27	32	DPDHVKGEGVVKKKKDKKKHEHGHEHG
KAJ7971926.1	27	32	EGHDNGNKDEKKKKKEKKKHEDGQDHG
RZS09437.1	27	32	GEEHGEEEKKDKKKKKKEKKKHGEEHH
KAE9619818.1	28	23	DGHEKGEKKKKKDKKEKKDKKHGEHGHG
PNX74794.1	28	23	GEDHESKGKGEKKDKKKKDKKKKEHGHD

XP_047320797.1	28	23	SDSHNKGEKNKEKKKKEGHERGKGHGHD
KAK2638285.1	29	19	DGGPQKGYDGEDKKKKKKDKKKKKEGHEH
KAI5659295.1	29	19	GGESGEHKGEEKKKKKKEKKKHGEGHDHH
XP_058204280.1	29	19	GGGTGGTQDHENKGEKKKKKKEKKKKHEGG
ABR25782.1	30	15	DHGDGGEHKEKKDKKKKKEKKHGEEGHHHD
BAA24979.1	30	15	DHGDGGEHKEKKDKKKKKEKKHGEEGYHHD
CAH9127439.1	30	15	EGGEKQGKDKKDKKKKKEKKSGHGHGHEHG
KAF7044537.1	31	14	DGKKDGDHKEKKDKKKKKDKKHGEGHKDDDG
KAH7571671.1	31	14	QGGQGGHEQGHGGVEKKKKEKKKKKEHGDKH
AAT76303.1	31	14	VGGAGGAGHENKGGEKKKKKDKKKHEDGHDH
NP_175843.1	32	6	GEGKSHDGEGKSHDGEKKKKKDKKEKKHHDDG
XP_030463629.2	32	6	GHGGDHHDKEGHEKKDKKDKKKKKDKKKHEHG
XP_030526053.2	33	8	HGDHHHDKEGHEKKDKKKKKEKKKHEHGHEHGH
CAA7388142.1	33	8	KGEGEGEGEGEKTEKKKKEKKKKKDKKEDEEKD
XP_017246606.1	34	5	DKSGDHHGHGHGHGHGDGKKKDKKKKKHEDGHKH
XP_066371966.1	34	5	EHGEKSGEHKDKDHKEKKDKKKKEKKHGEGHDHD
APJ36639.1	35	6	DGHGDGKKDGDHKEKKDKKKKKDKKHGEGHKDDDG
KAF8684364.1	35	6	EHGDKSGDHKDDKDHKEKKDKKKKKEKKHGEGYHD
XP_012828713.1	36	1	GDEGESGECKGEKKDKKKKDKKEKKKHCDGDGEDKH
KAF1001578.1	37	2	GSSEDKKKDEHYGDEKSHDKKDKKKKKDGHEKKKKDG
NP_001150115.2	38	3	EHGDKSGDHKDKDHKEKKDKKKKKEKKHGEGHDHGDGD
KAJ6795074.1	39	1	GGEHKEGEKKKHDGEKKKHDDGEKKKKKKDKKKHGDQHH
KAK8942396.1	42	1	GEKGEKEKNHGDKKEKKEKEKNHGDDKDKKKKKDKKEGKHHG
XP_039017539.1	44	1	DKDDDDKHHKKDKDDKHHKKDKDGHGSDGEKKKKKKGKKKHKDG
XP_039006091.1	46	2	EKEEDDKHHKKEKVKDDKHHKKDKDGPDNDGEKKKKKKGKKKHEDG
XP_062215072.1	47	2	EQGDKSGEHKDKSGEHKDKSGEHKDKDNKEKKDKKKKKDKKHGEGHD

XP_039063736.1	55	1	DKDNDDKHHKKDKDKDDKHHKKDKDDKHHKKDKDDHGSDGEKKRKNGKKKHGDG
KAH1083101.1	58	1	DDDRGRDRRRDSSRGRDSSREHHRDRDHDSDRESDRDSDGGSDDEKKKKKKKGKKKKD
MBA0742274.1	60	1	DDDRGRDRRRDSSRGRDSSRDREHHRDRDRDSDRESDRDSDGGSDDEKKKKKKKGKKKKD
MBA0770560.1	62	1	DDDRGRDRRRDSSRDRDHHRDSSREHHRDRDRDSDRESDRDSDGGSDDEKKKKKKKGKKKKD
KZV14150.1	116	1	EGGHEDDGEKKKKKEKKKHEQGYEHDDSSVRVAYTQGLLCPSLLMIIQVVQHATHLLAYKYPGHGRIYNLLIPRGQT<br>FSSPEDKASHPHGEGGHEDDGEKKKKKKEKKKHEQGYEH
KAK9017633.1	168	1	GDDDHDHDRKEHKKKEGEMMDKTKSKFHDDDDNDHERKEHKKKEGKMMDKTKDKFHDDDDDHDHKEHKKKKDKEYKK<br>EGGTVDKTKGKFHDDDDDHDHKEHKKKKSKEHKKERGMMDTIKDKFHDDDDDDRDRDRDRRRDNDSDHDSDRGSDGE<br>CKKKKKGKKKYRDD
XP_012836029.1	196	1	DDVTESWKRRYGYDRVNDDNDLPIIEAKATDEVGKDPFAERRKEKKGKVDKQEKNRLHNLKQANKVGALPSHIQLAA<br>TSLPITGTQSMPKKISKDELQHVAGMASTATASGGKFDKKLPGEKAPKHEKKYRKFLPAVEGSGMGALEKQQNENVL<br>NKLMSKHSHEILNVQKAVDMYNVKSDKKRKGGGGGGQQKKQW

Figure S1. Multiple sequence alignment of selected reticulon-like sequences in land plants. K-like segment outlined in red, S segment-like region outlined in green.

```
XP_044448329.1 reticulon-like protein B1 isoform X1 [Triticum aestivum]
KAA3475865.1 reticulon-like protein B3 [Gossypium australe]
ACN40158.1 unknown [Picea sitchensis]
XP_024437525.2 reticulon-like protein B5 [Populus trichocarpa]
CAA3014941.1 reticulon B2 [Olea europaea subsp. europaea]
NP_176592.1 Reticulan like protein B3 [Arabidopsis thaliana]
XP_006842617.1 reticulon-like protein B5 [Amborella trichopoda]
XP_023738480.1 reticulon-like protein B1 [Lactuca sativa]
PIA34304.1 hypothetical protein AQUCO_03800119v1 [Aquilegia coerulea]
XP_010671578.2 reticulon-like protein B4 [Beta vulgaris subsp. vulgaris]
```

```
CLUSTAL O(1.2.4) multiple sequence alignment. (Madeira F, Madhusoodanan N, Lee J, et al. The EMBL-EBI Job Dispatcher
sequence analysis tools framework in 2024. Nucleic Acids Research. 2024 Jul;52(W1):W521-W525. DOI: 10.1093/nar/gkae241.
PMID: 38597606; PMCID: PMC11223882.)
```

```
XP_044448329.1      MSRVFFVRILKNKRERYYKNRDNNPRSGEGRGVHTLAREGMMSESEEHGSLLEKIGDKIG    60
KAA3475865.1        -----------------------------------------------MVEHEESLMEKIA    13
ACN40158.1          ----------------------------------------MGEHPEEAASFLDSVMEKVA    20
XP_024437525.2      --------------------------MQKNEHTHTLKDPSMAEHEGQRESAAESLVDKIT    34
CAA3014941.1        -----------------------------------------MADHHKEEEKKEELMEKIS    19
NP_176592.1         --------------------------------------------MAEEHKHEESIMEKIS    16
XP_006842617.1      ----------------------------------------MPEGTDESGSHAESIMEKIK    20
XP_023738480.1      -------------------------------------------MSDHEDHKSESLMEKIS    17
PIA34304.1          -------------------------------------------MVVEHVEHSESLMEKIK    17
XP_010671578.2      -----------------------------------------MSDHAEEHHESSSLMEKIS    19
                                                                    ..: :*: 

XP_044448329.1      DKIHEFKKDSSSSDSDDDK------------------------------------KSHKS    84
KAA3475865.1        EKIHGHNSSSDSDDDKPS----------------------------------------KT    33
ACN40158.1          EKLPGHDSSSSDSDDEKSKK---------------------------PESKKPEPSSISS    53
XP_024437525.2      EKINGHDSSSSSDSDNEK------------------------------------FDAVKS    58
CAA3014941.1        DKFHGHDSSTSDDEDE--K-----------------------------------KSSTKA    42
NP_176592.1         EKIHGHDDSSSSSSDSDDDK---------------------------------NSASLKT    43
XP_006842617.1      EKIHGEDDSSSSSSSSDSE-----------------------------TEKPSLSAAKA    50
XP_023738480.1      DKIHGKDDSSSSSDSDDDK-----------------------------------ISAMKS    42
PIA34304.1          EKIHGDNSSDSDNDNKKEKPHSSSSSSS------------------------PSPSTVKS    53
XP_010671578.2      DKIHGHDSSSSSSDEESDKKKSSSSETPSVPAAAHVPPPEVVPATEKAEAPASPSSSTSS    79
```

```
                     :*:   ... ......                                          .:

XP_044448329.1       KKKHLFGRKHPLHNVLGGGKAADLVMWRDKQKSGSILGGVTVIWLLFEGIGYHLLTFLCH    144
KAA3475865.1         SVFRPFGRERPVHRVFVGGKHMLQKLF-------------------FSFLKFLFFFFLKF    74
ACN40158.1           SVKRLFGREKPVHTLFGGGKSADVILWRNKQTSAGVLAGATVMWLLFEWLGYYLLTLICH    113
XP_024437525.2       KIFRLFGREKPVHKVLGGGKPADVFLWRNTKISAGVLGGATTVWVLFELLGYHLITLVCY    118
CAA3014941.1         KVHRIFGREKSVHKTLGGGKLADVFLWRDKKVSAGVLGGATAIWVLFDVLEYNLLTLVCH    102
NP_176592.1          KIYRLFGREQPLHKLFGGGKPADIFLWRNKKVSGGVLGAATVSWILFELLEYNLLTLFGH    103
XP_006842617.1       KVNRLFGRQKPVHSVLGGGKPADVLLWRNKQISAGVLVGVTVIWLLFEWLGYHLLTLICH    110
XP_023738480.1       KVFRMFGREKPVHNVFGGGKPADVLLWKDKKVSGGILGGVSIIWFLFEVMEYHLLTLVCH    102
PIA34304.1           AINRLFGRQKPVHKVFGGGKPADVFLWRNKKISAGVLGGATAIWVLFELLEYHLLTLVCH    113
XP_010671578.2       KIYRLFGRQKPVHKVLGGGKPADVLLWRNKKISASALGVATVIWFLFELLEYHLLTLLCH    139
                        : ***:: :*  : ***     ::                   *. : : :: :. .

XP_044448329.1       LLIIFLTVSFVWSNAASFINRSPPKFPEVILSETQCLIIAHVLR----------------    188
KAA3475865.1         FLWSLKSILFVCANVEIVLNRSPTRIPEVQIPKDPVLECAQSLR----------------    118
ACN40158.1           VLILGITILFVWSNASAFINKQPVRIPQISFSEQLFQDVASALR----------------    157
XP_024437525.2       LSIFSLAVLFLWSNTSDFLNKPPPKIPEVVLPEKCVREVASVLR----------------    162
CAA3014941.1         GLILALVILFLWSNASTFIKKSPPRIPDVNIPEKPVLKFASAWRIEINRAFSTLREIASG    162
NP_176592.1          ISILALAVLFLWSSASTFIHKSPLHIPEVHIPEDVVLQLASGLR----------------    147
XP_006842617.1       SLVLSLSLLFLWSNASALINKSPPKFPEINLSEDVFVSIAVSLT----------------    154
XP_023738480.1       SLILTLAILFLWSNASAFINKSPPKIPQVVIPEKPVLEIVSSLR----------------    146
PIA34304.1           CLILVLALSFLWSNAATFVNKSPPQIPNLCLPEKQVVEVASALS----------------    157
XP_010671578.2       ILIFTLAVLFLWSNASAFIHKAPPRIPDVHIPDNALLEFISVLR----------------    183
                            : *: :..  .::: * ::*:: : .

XP_044448329.1       ------------------------------------------------------------    188
KAA3475865.1         ------------------------------------------------------------    118
ACN40158.1           ------------------------------------------------------------    157
XP_024437525.2       ------------------------------------------------------------    162
CAA3014941.1         RDLKKFLSVSGPSTSNLEAITNLVISSAFLANCCDFLTVVYISTVFLCTVPVLYEKYGDK    222
NP_176592.1          ------------------------------------------------------------    147
XP_006842617.1       ------------------------------------------------------------    154
XP_023738480.1       ------------------------------------------------------------    146
PIA34304.1           ------------------------------------------------------------    157
XP_010671578.2       ------------------------------------------------------------    183
```

```
XP_044448329.1        -----------------------------------------KEINEAFITLQSVASGKDL    207
KAA3475865.1          -----------------------------------------FEINCAFSVLQDIASGRYL    137
ACN40158.1            -----------------------------------------FEINRLFTVIHNVASGRDL    176
XP_024437525.2        -----------------------------------------IEINRGFAVLRDIASGRDL    181
CAA3014941.1          VDSFAEKAISELKKQYAVLDDKVLNVNIPEKPVLKFASAWRIEINRAFSTLREIASGRDL    282
NP_176592.1           -----------------------------------------IEINRGFTVLRDIASGRDL    166
XP_006842617.1        -----------------------------------------YELNCVFATLRDVASGRDL    173
XP_023738480.1        -----------------------------------------IELNNGFAAIRDIASGKDL    165
PIA34304.1            -----------------------------------------KEINCAISVLRDVASGRDV    176
XP_010671578.2        -----------------------------------------FEINRALSVLRDIASGKEL    202
                                                                *:*  : .::.:***: :

XP_044448329.1        KTYLKSIGVLWFISIIGGCFSFLTLSYTIFLMAYTLPMLYEKYEDEVDVVGEKALIELKK    267
KAA3475865.1          KKFLSVIASLWVLSIVGSWCNFLTLFYIVFILLHTVLVLYEKYEDKVDPFAEKAMHEIKK    197
ACN40158.1            KKFLMVVAGLWILSVIGSWANFLTLFYVAFVFAHTVPVLYEKYEDQVDAFTFKAMDEAKK    236
XP_024437525.2        KKVLAVIAGLWVLSLIGSCINILTLLYISFVLLHTVPVLYEKHEDQVDAYSEKAWIEIKK    241
CAA3014941.1          KKFLSVIAGLWILSIIGSCCDFLTVVYISTVFLCTVPVLYEKYGDKVDSFAEKAISELKK    342
NP_176592.1           KKFLLVIAGLWVLSKVGSSCNFLTLIYIATVLLFTIPVLYEKYEDKVDDFGEKAMREIKK    226
XP_006842617.1        KKFLLVTAGLWVLSVVGSWCNFLTLFYIVFVILHTVPVLYEKHEDKVDFFAERALGEAKK    233
XP_023738480.1        KKFLSVIAGLWFVSIISNCYNFLTLVYILVVLLFTVPVVYDKYEDKIDPLAEKALIEIKK    225
PIA34304.1            KTFLFVIAGLWVLSIVGSWCNFVSLVYIAFVLLHTVPVLYEKYEDQVDTFAEKAMHEIKK    236
XP_010671578.2        KKFFRVIAGLWVVSVVGSWFNFLTLVYLLFVLLHTVPVLYEKYEDHVDAFAEKAAIELKK    262
                      *. :   . **.:* :..  .:::: *   ::  *: ::*:*: *.:*    :*  * **

XP_044448329.1        QYAVFDEKVLS----KIPMLADKKQH----    289
KAA3475865.1          QCAVLNTKFLRFRGD----G----------    213
ACN40158.1            HYGTFDAKVLS----KIPRGPLKDKKF---    259
XP_024437525.2        QYAVFDEKYLS----KIPKGALKGEKKD--    265
CAA3014941.1          QYAVLDDKVLS----KIPRGPSKDKKR---    365
NP_176592.1           QYVEFDVKVLSKVMSKIPKGAFAFIKKKD-    255
XP_006842617.1        QYRVVDAKFLQ----KIPKGPFKDKKLH--    257
XP_023738480.1        QYAVFDAKVLC----KIPRSMKELKAKKKV    251
PIA34304.1            QYAVFDAKVLS----KIPRGPSRDKKHL--    260
XP_010671578.2        QYAVADEKFLS----KIPRGPLKDKKSA--    286
```

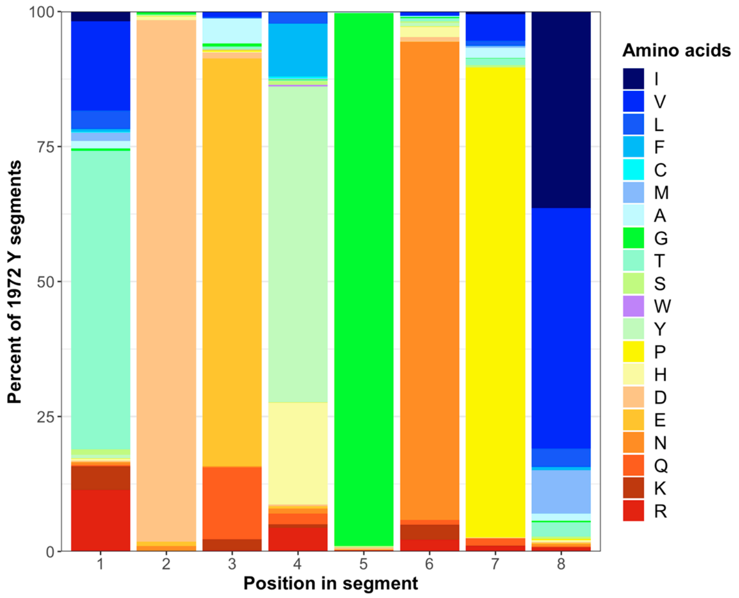

Figure S2. Amino acid frequencies by position in 1792 8-residue Y segments identified by Dhn Decoder.

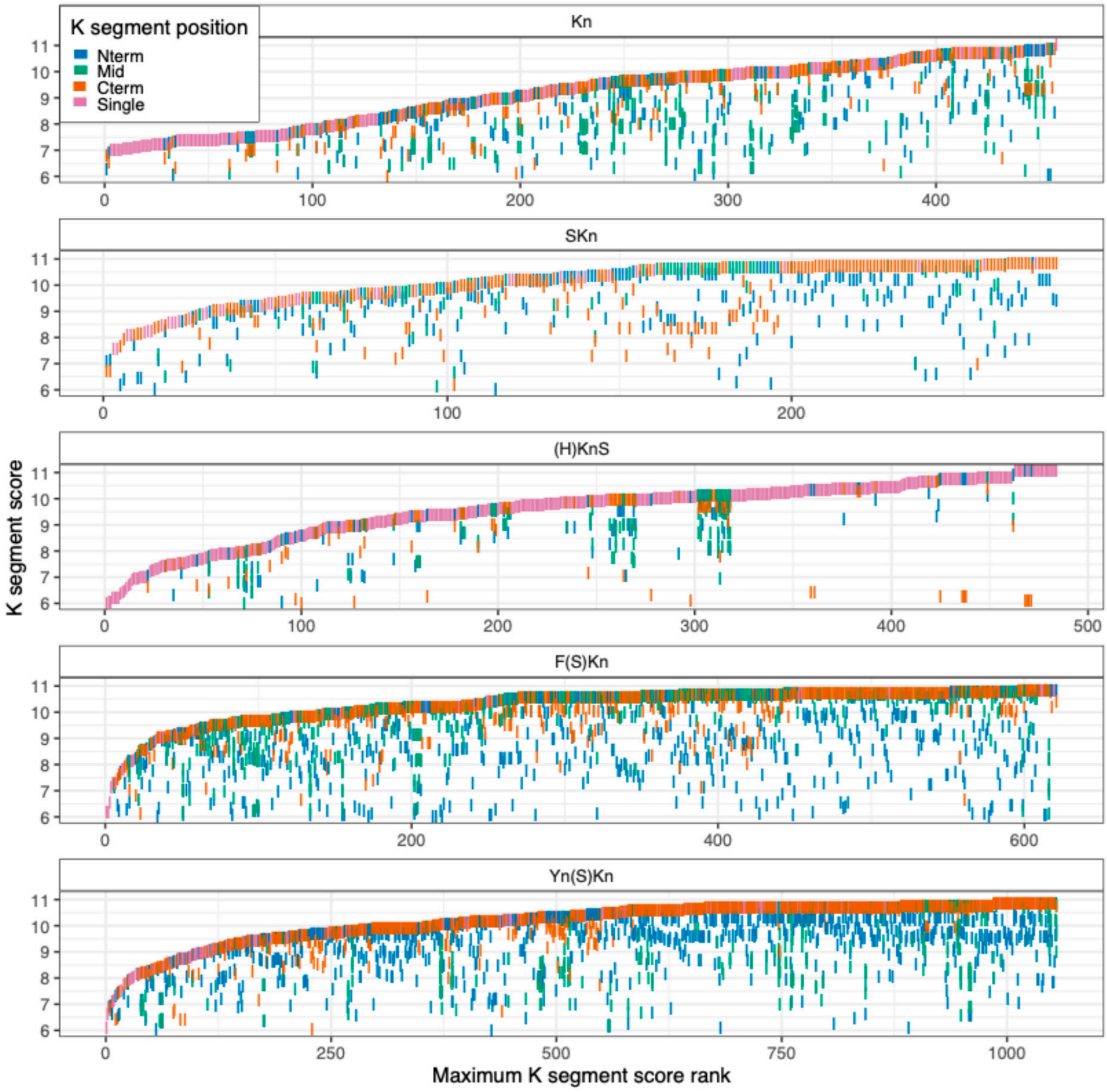

Figure S3. Dhn Decoder scores for K segments in N terminal, intermediate, and C terminal positions in Dhns with ≥2 K segments and for the single Ksegment in Dhns with only one. Dhns in each group are ranked by the maximum score in each protein.

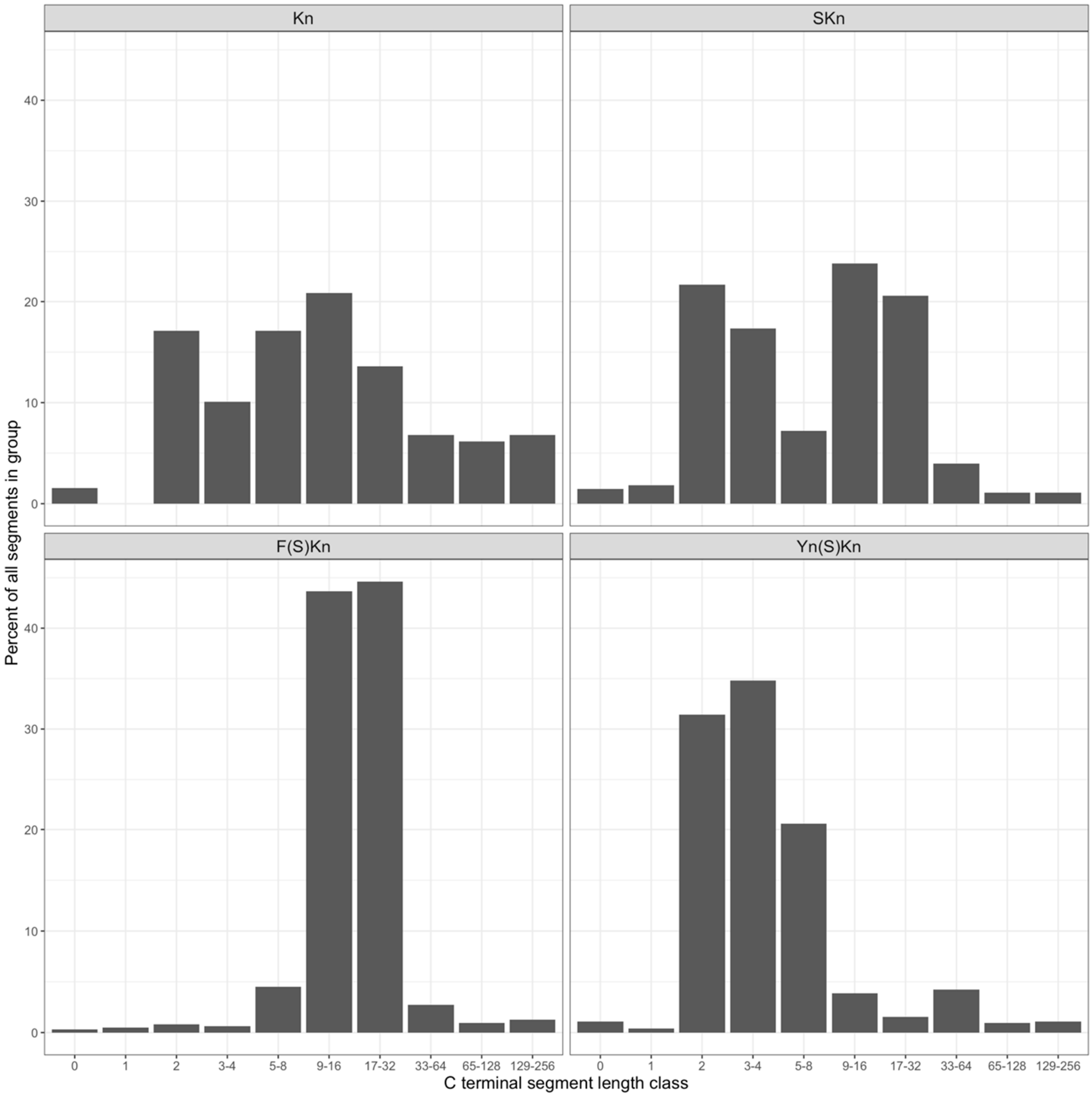

Figure S4. Lengths of C terminal segments following K segments in Dhn orders.
